# Effect of the Heteroaromatic Antenna on the Binding of Chiral Eu(III) Complexes to Bovine Serum Albumin

**DOI:** 10.1021/acs.inorgchem.0c01663

**Published:** 2020-08-10

**Authors:** Chiara De Rosa, Andrea Melchior, Martina Sanadar, Marilena Tolazzi, Alejandro Giorgetti, Rui P. Ribeiro, Chiara Nardon, Fabio Piccinelli

**Affiliations:** † Luminescent Materials Laboratory, Department of Biotechnology, University of Verona and INSTM - UdR Verona, Strada Le Grazie 15, 37134 Verona, Italy; ‡ Laboratory of Chemical Technologies, Polytechnic Department of Engineering and Architecture, University of Udine, via Cotonificio 108, 33100 Udine, Italy; § Applied Bioinformatics Laboratory, Department of Biotechnology, University of Verona, Strada Le Grazie 15, 37134 Verona, Italy

## Abstract

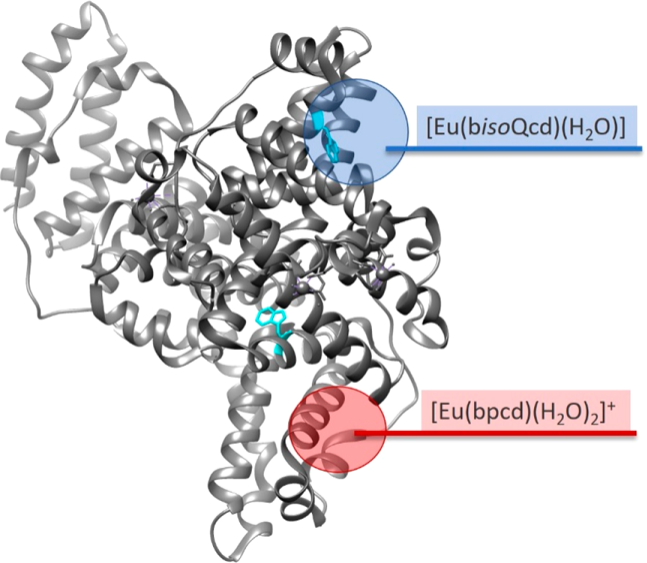

The cationic enantiopure (*R,R*) and luminescent Eu(III) complex [Eu(b*iso*Qcd)(H_2_O)_2_] OTf (with b*iso*Qcd = *N,N′*-bis(2-*iso*quinolinmethyl)-*trans*-1,2-diaminocyclohexane *N,N′*-diacetate and OTf = triflate) was synthesized and characterized. At physiological pH, the 1:1 [Eu(b*iso*Qcd)(H_2_O)_2_]^+^ species, possessing two water molecules in the inner coordination sphere, is largely dominant. The interaction with bovine serum albumin (BSA) was studied by means of several experimental techniques, such as luminescence spectroscopy, isothermal titration calorimetry (ITC), molecular docking (MD), and molecular dynamics simulations (MDS). In this direction, a ligand competition study was also performed by using three clinically established drugs (i.e., ibuprofen, warfarin, and digitoxin). The nature of this interaction is strongly affected by the type of the involved heteroaromatic antenna in the Eu(III) complexes. In fact, the presence of *iso*quinoline rings drives the corresponding complex toward the protein superficial area containing the tryptophan residue 134 (Trp134). As the main consequence, the metal center undergoes the loss of one water molecule upon interaction with the side chain of a glutamic acid residue. On the other hand, the similar complex containing pyridine rings ([Eu(bpcd)(H_2_O)_2_]Cl with bpcd = *N,N′*-bis(2-pyridylmethyl)-trans-1,2-diaminocyclohexane *N,N′*-diacetate) interacts more weakly with the protein in a different superficial cavity, without losing the coordinated water molecules.

## Abstract 2

The effect of the antenna moiety on the interaction of two new luminescent Eu(III) complexes with BSA was studied. Results show that the complexes can be conveniently exploited as optical probes for albumin serum proteins by means of opposite mechanisms (switch-on−off of the luminescent signal).

## Introduction

The optical sensing of biologically relevant species is often performed by lanthanide (Ln(III)) derivatives, mainly Eu(III) and Tb(III) complexes^1−6^ due to their peculiar properties, such as long Ln(III) luminescence lifetime and the possibility to exploit the so-called *antenna effect*. Thanks to the first peculiarity, one can use time-gated detection to mitigate the interference of background fluorescence originating from the biological sample and hence to isolate the Ln(III) luminescence. This advantageous feature has been exploited, for example, to obtain a high signal-to-noise (S/N) ratio by means of Eu(III)-based probes in the detection of intracellular pH^7^ as well as other important bioanalytes, such as bicarbonate,^8,9^ citrate,^10^ lactate,^11^ ATP,^12,13^ and vitamin C.^14^ Based on the *antenna effect,* the luminescence intensity of the metal ion can be considerably enhanced if the ligand is capable of strongly absorbing and efficiently transferring the UV excitation to the Ln(III) center. At the same time, high values of the emission quantum yield are required. High emission intensity is mandatory when emissive lanthanide complexes are used as tags in bioassays or as optical probes.^6^ In addition to optimal emission properties, a luminescent Ln(III) complex must interact selectively with a target bioanalyte in a complex matrix containing competing species, such as proteins. Among the main proteins in the biological fluids, serum albumin (SA), which represents 52% of the protein composition in the circulatory system, has been broadly studied in light of its very important physiological and pharmacological functions.^15^ For example, SA has a limited number of binding sites with high specificity,^16^ which play a crucial role in the transportation and delivery of a variety of endogenous and exogenous species.^17^

Human serum albumin (HSA) and bovine serum albumin (BSA) are the most extensively studied serum proteins. Changes of the albumin levels in blood and urine could be related to several disorders, including liver disease, neoplasia, nephrotic and diabetic syndrome, and severe dehydration.^15^ Serum albumin can bind lanthanide complexes,^18−20^ often leading to significant changes of the Eu(III) and Tb(III) luminescence features,^21−26^ as well as of the protein fluorescence.^27−31^ Such a piece of evidence offers the possibility to detect these proteins by means of optical spectroscopy techniques. Furthermore, since strong changes in the fluorescence spectra of the proteins are due to interactions close to the Trp residues, the regiochemistry can often be evaluated. In this regard, there are marked similarities between BSA and HSA in their composition and structure. In fact, although HSA contains two extra amino acid residues (total 585) compared to BSA, the carried-out protein sequence alignment (1AO6.pdb and 3V03.pdb for HSA and BSA, respectively; Figure S1) pointed out about 76% amino acid sequence identity. Both proteins consist of two identical chains, A and B, in turn subdivided into three homologous evolutionarily related domains (I, II, III) (Figure S2). Each chain is predominantly in the α-helix form (74%), but HSA has only one tryptophan residue (Trp-214, buried within the secondary structure), while BSA contains two, namely, Trp-213 (confined within a hydrophobic pocket of the protein in the domain II) and Trp-134 (located at the outer surface of the protein in the domain I).^32^ Thus, BSA can be used as a model for HSA, with the advantage of offering two structural probes (Figure S3).

Although the emission of BSA originates mainly from the two tryptophan residues, tyrosine and phenylalanine side chains give a partial contribution to the overall BSA fluorescence.^33^ Therefore, significant changes of the fluorescence intensity of the BSA protein could be ascribed to interactions close to the aforementioned domains (I and II), whereas small changes can be due to interactions occurring in other protein sites, also near tyrosine and phenylalanine residues. In addition, the fluorescence intensity is also sensitive to small modifications of the local protein secondary structure.^34^

**Figure 1:**
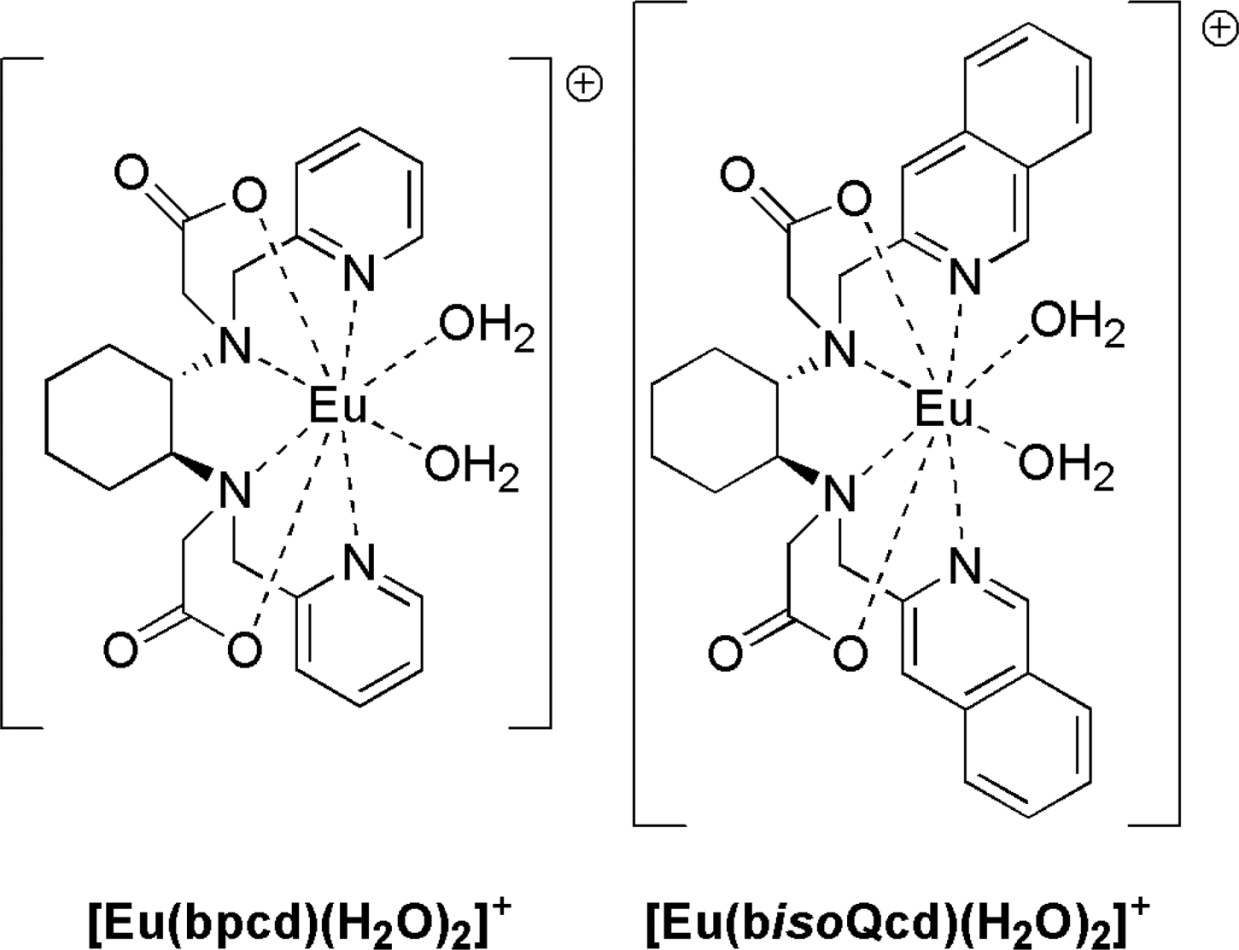
Molecular structure of the Eu(III) complexes investigated here, where bpcd and b*iso*Qcd stand for *N,N′*-bis(2-pyridylmethyl)-trans-1,2-diaminocyclohexane *N,N′*-diacetate and *N,N′*-bis(2-*iso*quinolinmethyl)-trans-1,2-diaminocyclohexane *N,N′*-diacetate, respectively. For the sake of clarity, the type of counterion—Cl^–^ in the case of [Eu(bpcd)(H_2_O)_2_]^+^ and CF_3_SO_3_^–^ in the case of [Eu(b*iso*Qcd)(H_2_O)_2_]^+^—is omitted.

In this work, after the synthesis and physicochemical characterization of two cationic water-soluble Eu(III) luminescent complexes, the interaction with BSA was studied by means of several complementary experimental techniques (emission spectroscopy, isothermal titration calorimetry) and biomolecular simulations to obtain information on the affinity, specificity, and structural details of the complex/protein adduct. The investigated complexes (Figure 1) differ in the nature of the heteroaromatic antenna (pyridine vs isoquinoline), thus being labeled as [Eu(bpcd)(H_2_O)_2_]Cl and [Eu(b*iso*Qcd)(H_2_O)_2_]OTf, respectively. In both cases, the Eu(III) ion is 8-fold coordinated by one ligand molecule, contributing with six donor atoms, and two water molecules. For sensing purposes, these two molecules can be easily displaced by the target analyte. Since the chirality plays an important role in the interaction with biomolecules, the configuration of the two stereogenic carbon atoms of the ligands was fixed (*R,R* stereochemistry) so to study enantiopure complexes.

**Figure 2:**
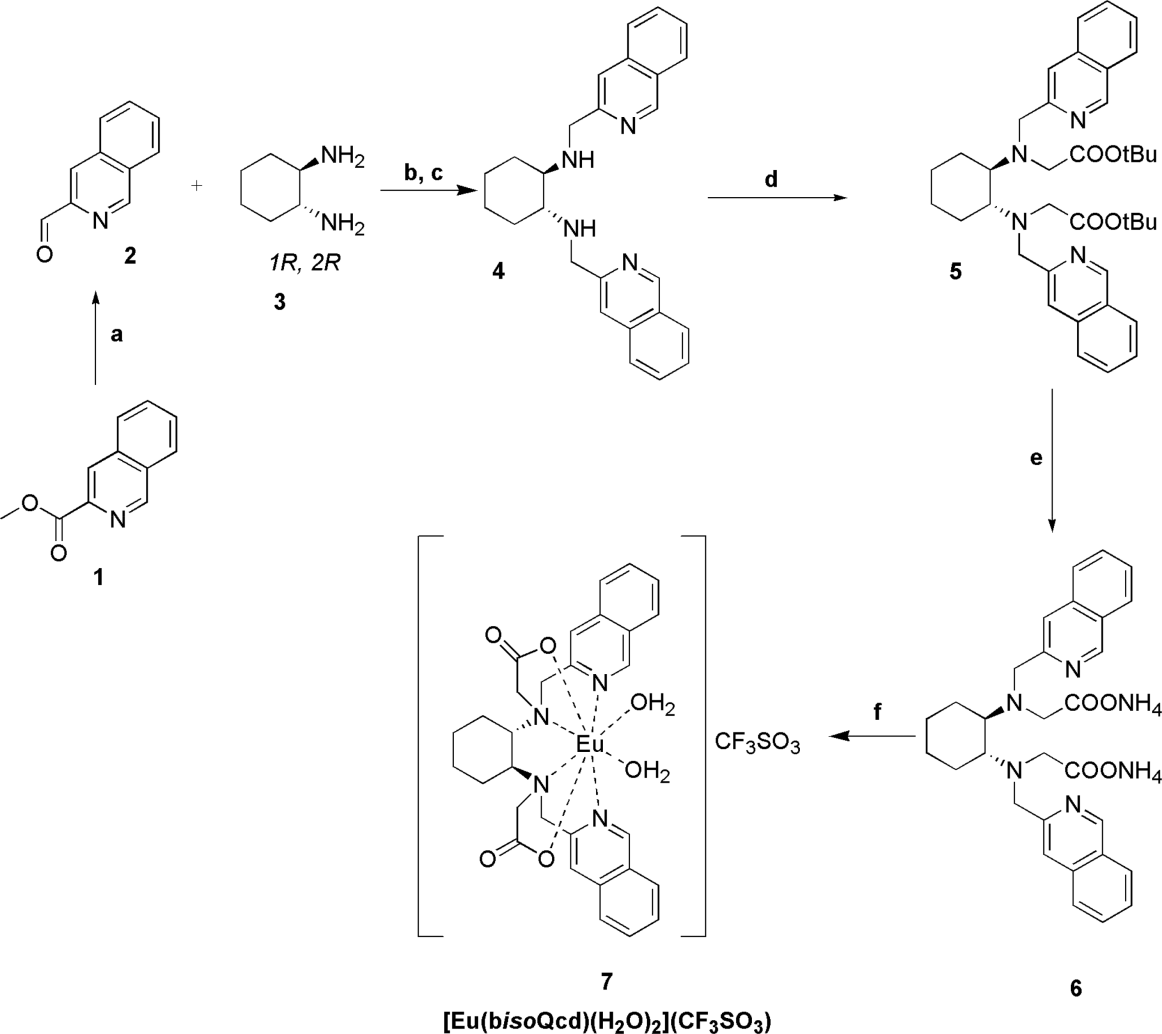
Synthetic procedure for the new [Eu(b*iso*Qcd)(H_2_O)_2_]CF_3_SO_3_ compound. (a) Diisobutylaluminum hydride (DIBAL-H) 1 M in toluene 1.7 equiv, −78 °C; (b) Isoquinoline-3-carbaldehyde ∼1.1 equiv, absolute ethanol, room temperature, 12 h; (c) NaBH_4_ 1.8 equiv, MeOH, room temperature, 5 h; (d) *tert*-butyl bromoacetate 2.5 equiv, K_2_CO_3_ 2.7 equiv, MeCN, room temperature, 12 h; (e) HCl 6 M aq. 80 °C, 12 h; (f) Eu(CF_3_SO_3_)_3_ 1 equiv, 2-propanol, room temperature, 12 h.

The protocol for the synthesis of the new [Eu(b*iso*Qcd)(H_2_O)_2_]CF_3_SO_3_ complex is reported in Figure 2.

## Materials and Methods

All commercially available reagents were used as received from suppliers. Solvents (Sigma-Aldrich) were dried when required using an appropriate drying agent. Reactions requiring anhydrous conditions were carried out using Schlenk-line techniques under an atmosphere of dry argon. Water refers to high purity H_2_O obtained from the “Millipore Elix 10” purification system. Eu(CF_3_SO_3_)_3_ (Aldrich, 98%) was stored under vacuum for several days at 80 °C and then transferred to the glovebox. All other chemicals were purchased from Alfa Aesar. Thin-layer chromatography was carried out on neutral alumina plates (Fluka Analytical) or silica plates (Sigma-Aldrich) and visualized under UV lamp (254 nm). The cationic exchange chromatography was performed on SCX cartridges (1 g) purchased from “Agilent Technologies-Sample Prep solutions”. 4-Morpholinepropanesulfonic acid (MOPS) buffer (purity 99%) was dissolved in 0.9% w/v NaCl water solution. The pH value was corrected to the physiological value (7.4) by dropwise addition of freshly prepared NaOH 10 M. Stock solution of BSA (purity ≥99%, purchased from Sigma-Aldrich) was freshly prepared and used (2 × 10^–4^ mol L^–1^) by dissolving the protein in MOPS buffer. The solution was kept in the dark at 4 °C before use.

### Synthesis

The [Eu(bpcd)(H_2_O)_2_]Cl complex (Figure 1) was synthesized as reported in the literature.^35^ Its analogue [Eu(b*iso*Qcd)(H_2_O)_2_](CF_3_SO_3_) (Figure 1), bearing isoquinoline heteroaromatic pendants, instead of pyridine ones, was obtained following the synthetic protocol reported in Figure 2.

### Isoquinoline-3-carbaldehyde (**2**)

Under inert atmosphere, DIBAL-H 1 M in toluene (54.5 mL, 55 mmol) was added dropwise to a stirred solution of the commercially available methyl ester **1** (6 g, 32 mmol) in anhydrous toluene (200 mL) at −78 °C. The mixture was stirred at this temperature for 50 min and then allowed to reach 0 °C. Under argon flow, 1 M HCl (48 mL) was dropwise added and the resulting suspension was filtered through a Celite pad. The filtrate was diluted with water (350 mL) and extracted with ethyl acetate (3 × 350 mL). The combined organic phases were washed with saturated aqueous NaCl solution, dried over Na_2_SO_4_, and the solvents were evaporated *in vacuo* to give 3.44 g of a reddish solid, which was used in the next step without further purification. Yield: 68%, purity: 80% (determined by ^1^H NMR technique). ^1^H NMR (CDCl_3_) δ (ppm) 7.78 [2H, t, *J* = 7.74 Hz]; 7.99 [1H, d, *J* = 7.74 Hz]; 8.05 [1H, d, *J* = 7.74 Hz]; 8.36 [1H, s]; 9.35 [1H, s]; 10.25 [1H, -CHO]. ^13^C NMR (CDCl_3_): 121.78 (CH-Ar), 127.79 (CH-Ar), 128.62 (CH-Ar), 130.21 (CH-Ar), 130.52 (-C-Ar), 131.44 (CH-Ar), 135.24 (-C-Ar), 146.81 (-C-CHO), 153.25 (CH-N-CHO), 193.35 (CHO).

### *N*,*N*′-Bis-isoquinolin-3-ylmethyl-cyclohexane-1,2-diamine (1R, 2R) (**4**)

Purchased *trans*-1(*R*),2(*R*)-diaminecyclohexane (**3**) (1.55 g, 13.56 mmol) was added at RT to a stirred solution of isoquinoline-3-carbaldehyde (2.34 g, 14.9 mmol) in anhydrous EtOH (145 mL). The yellowish reaction mixture was stirred to room temperature for 12 h; then, upon cooling at ∼0 °C sodium borohydride (0.923 g, 24.4 mmol) was directly added (one pot) to the mixture to get a clear reddish solution. After 5 h, the reaction mixture was quenched with water and the product was extracted with dichloromethane. The collected organics were washed with brine solution and dried over sodium sulfate. Upon removal of the solvent under reduced pressure, 3.47 g of a yellowish oil was obtained (yield: 65%, purity: 80%, determined by ^1^H NMR technique).

^1^H NMR (CDCl_3_) δ (ppm) 9.17 (s, 2H, isoquinoline), 7.88 (m, 2H, isoquinoline), 7.74 (d, *J* = 8.27 Hz, 2H, isoquinoline), 7.64 (s, 2H, isoquinoline), 7.61 (m, 2H, isoquinoline), 7.51 (m, 2H, isoquinoline), 4.16 (d, *J*_GEM_ = 13.70 Hz, 2H, methylene), 4.06 (d, *J*_GEM_ = 13.70 Hz, 2H, methylene), 2.51 (d, 2H, “CH” cyclohexane), 2.22 (m, 2H, cyclohexane), 1.77 (m, 3H, cyclohexane), 1.14–1.41 (m, 2H, cyclohexane), 1.00 (m, 1H, cyclohexane). ^13^C NMR (CDCl_3_) δ (ppm) 25.21 (2 CH_2_), 25.39 (CH_2_), 31.38 (CH_2_), 52.42 (CH), 55.35 (CH), 57.60 (2 CH_2_), 118.02 (−2CH Ar), 126.42 (−2CH), 126.78 (−2CH), 127.54 (−C), 127.62 (−C−), 130.45 (−2CH), 136.40 (−2C−), 152.22 (−2CH), 153.59 (−2CH), 153.85 (−C–N), 154.35 (−C–N).

### {[2-(*tert*-Butoxycarbonylmethyl-isoquinolin-3-ylmethylamino)-cyclohexyl]-isoquinolin-3-ylmethylamino}-acetic acid *tert*-butyl ester (1R, 2R) (**5**)

Amine **4** (0.844 mmol) was dissolved in a mixture of anhydrous acetonitrile (13 mL) and anhydrous potassium carbonate (0.315 g, 2.28 mmol) under inert condition (argon). Then, a solution of *tert*-butyl 2-bromoacetate (0.312 mL, 2.11 mmol) in anhydrous acetonitrile (5 mL) was added dropwise over 5 min. After stirring over 12 h at room temperature, dichloromethane was added, and the reaction mixture was washed with brine solution. The organic phase was evaporated under reduced pressure to give 0.588 g of a yellowish oil. The product was purified by chromatographic column on activated neutral alumina (Al_2_O_3_) using a mixture of cyclohexane (Cy) and ethyl acetate (AcOEt) for the elution of the product (Rf = 0.35; Cy:AcOEt 7:3 to 1:9). In order to completely collect the product, a mixture of AcOEt and methanol was exploited (AcOEt:MeOH 9:1). 380 mg of a yellowish solid were obtained. Yield = 72%. ^1^H NMR (CDCl_3_) δ (ppm) 9.04 (s, 1H), 8.10 (s, 1H), 7.98 (m, 1H), 7.88 (d, *J* = 7.39 Hz, 2H), 7.74 (m, 3H), 7.56 (m, 4H), 4.26 (m, 6H), 4.04 (m, 1H), 3.62 (m, 1H), 2.79 (m, 1H), 2.31 (m, 2H), 1.83 (m, 3H), 1.46 (s, 18H), 1.28–1.02 (m, 4H).

### {[2-(Carboxymethyl-isoquinolin-3-ylmethylamino)-cyclohexyl]-isoquinolin-3-ylmethylamino}-acetic acid (1R, 2R) (**H**_**2**_**bisoQcd**, ligand **6** as an ammonium salt)

**5** (crude, ∼5.15 mmol) was dissolved in HCl aq (6 M, 80 mL) and stirred for 12 h at 80 °C. The volume of the reaction mixture was halved under reduced pressure and the product was purified by cationic exchange chromatography SCX (eluent: NH_3_ 3 M in MeOH) yielding 1.55 g of the desired product as a brownish solid. Yield: 55%. ^1^H NMR (DMSO) δ ppm: 9.31–9.21 (m, 2H), 8.15–8.05 (m, 2H), 8.01–7.89 (m, 2H), 7.80–7.71 (m, 2H), 7.69–7.58 (m, 2H), 4.96–4.71 (m, 1H, CH_2_), 4.30–3.63 (m, 4H, CH_2_), 3.56–3.21 (m, 3H, CH_2_), 2.30–1.82 (m, 3H, Cy), 1.76–1.50 (m, 3H, Cy), 1.37–0.96 (m, 4H, Cy). UV–vis spectrophotometry (methanol): ε(322 nm): 5174 M^–1^ cm^–1^. ESI-MS (Scan ES+; *m*/*z*): 573 (100%) = [NaKHL]^+^; L = b*iso*Qcd^2–^.

The triflate of the cationic complex [Eu(b*iso*Qcd)(H_2_O)_2_]^+^ (complex **7**) was synthesized as follows. Ligand **6** (60 mg, 0.110 mmol) was dissolved in hot (55 °C) 2-propanol (4 mL). Upon cooling, Eu(III) trifluoromethanesulfonate 98% (66 mg, 0.110 mmol) was added portion-wise, and a yellowish suspension was formed. After neutralization with KOH 2 M aq. (pH ∼7), the reaction mixture was stirred at room temperature for 12 h. The suspension was centrifuged, the filtrate was concentrated under reduced pressure, and the resulting solid (105 mg) was purified by dissolution in methanol, followed by precipitation in diethyl ether. Upon centrifugation, 28.6 mg of the desired complex was collected as a beigeish solid. Yield: 32%. UV–vis spectrophotometry: ε (323 nm): 4510 M^–1^ cm^–1^ (water); ε (323 nm): 7251 M^–1^ cm^–1^ (methanol). ESI-MS (Scan ES+; *m*/*z*) in methanol: 663 (100%); 661 (90%); ([Eu(b*iso*Qcd)]^+^). 695 (32%); 693 (21%); ([Eu(b*iso*Qcd)(CH_3_OH)]^+^). Elemental Anal. Calcd for C_31_H_30_EuF_3_N_4_O_7_S·(H_2_O)_2_ (MW 847.6): C, 43.93; H, 4.04; N, 6.61; O, 16.99; S, 3.78; found: C, 43.83; H, 3.97; N, 6.52; O,17.08; S, 3.88.

### ^1^H NMR Spectroscopy

Nuclear magnetic resonance (NMR) experiments were performed at 298.15 K using a 600 MHz Bruker Avance III spectrometer equipped with a triple resonance TCI cryogenic probe. Spectra were usually recorded in CDCl_3_ and, unless otherwise stated, chemical shifts are expressed as ppm and referenced to the internal standard tetramethylsilane (TMS). One-dimensional NMR spectra were recorded with 32 scans and a spectral width of 12019 Hz. All spectra were manually phased and baseline corrected using TOPSPIN 3.2 (Bruker, Karlsruhe, Germany). Chemical shift, multiplicity (s, singlet; d, doublet; t, triplet; m, multiplet; b, broad), coupling constants and integration area are reported.

### ESI-MS

Electrospray ionization mass spectra (ESI-MS) were recorded with a Finnigan LXQ Linear Ion Trap (Thermo Scientific, San Jose, CA, USA) operating in positive ion mode. The data acquisition was under the control of the Xcalibur software (Thermo Scientific). A MeOH solution of sample was properly diluted and injected into the ion source at a flow rate of 10 μL/min with the aid of a syringe pump. The typical source conditions were: transfer line capillary at 275 °C; ion spray voltage at 4.70 kV; sheath, auxiliary, and sweep gas (N_2_) flow rates at 10, 5, and 0 arbitrary units, respectively. Helium was used as the collision damping gas in the ion trap set at a pressure of 1 mTorr.

### Elemental Analysis

Elemental analyses were carried out by using an EACE 1110 CHNOS analyzer.

### UV–vis Spectrophotometry

Room temperature electronic spectra were acquired by a Cary 60 UV–vis spectrophotometer, equipped with a xenon lamp single source (80 Hz), Czerny–Turner monochromator, and a photomultiplier (dual silicon diode detectors); scan rate: 300 nm/min in the 200–800 nm range.

### Luminescence and Decay Kinetics

Room temperature luminescence was recorded by a Fluorolog 3 (Horiba-Jobin Yvon) spectrofluorometer, equipped with a Xe lamp, a double excitation monochromator, a single emission monochromator (mod. HR320), and a photomultiplier in photon counting mode for the detection of the emitted signal. All the spectra were corrected for the spectral distortions of the setup.

In decay kinetic measurements of Eu(III), a Xenon microsecond flashlamp was used and the signal was recorded by means of a multichannel scaling method. True decay times were obtained using the convolution of the instrumental response function with an exponential function and the least-squares-sum-based fitting program (SpectraSolve software package).

The decay kinetics of the protein fluorescence was measured at 298 K, using a Chronos BH ISS Photon Counting instrument with picosecond laser excitation at 280 nm operating at 50 MHz. Fluorescence decays were then globally fitted with exponential functions using Glotaran ver. 1.5.1 software.^36^

### Potentiometric and Spectrophotometric Titrations

Stock solutions of NaOH and HCl were prepared by diluting 1.0 M standard solutions (Fluka Analytical) in ultrapure water (>18 MΩ·cm, ELGA Purelab UHQ). The ionic strength of all solutions was adjusted to 0.1 M with appropriate amounts of NaCl (Riedel-de Häen). Stock solutions of Eu(III) (80 mM) were prepared by dissolving the chloride salt (Sigma-Aldrich). The lanthanide content in the stock solutions was determined by titration with EDTA and xylenol orange as an indicator in acetate buffer.^37^ Free acid concentration in lanthanide solutions were checked by Gran’s method.^38^

Protonation constants of the b*iso*Qcd ligand was determined by acid–base potentiometric titrations. The titration cell was maintained at constant temperature (*T* = 298.2 ± 0.1 K) with a circulatory bath and under an argon radial flux. The electromotive force (emf) data were collected by using a computer-controlled potentiometer (Amel Instruments, 338 pH Meter) connected to a combined glass electrode (Metrohm Unitrode 6.0259.100). The electrode was calibrated before each experiment by an acid–base titration with standard HCl and NaOH solutions. The carbonate impurity in solution was checked by the Gran’s method. Titrations were performed by adding NaOH or HCl to ligand solutions (total ligand concentration, *C*°_L_ = 0.72 mM) by a computer-controlled buret (Metrhom Dosimat 765). At least 100 points were collected for each titration which were afterward processed with the Hyperquad^39^ program to calculate the protonation constants.

The formation constants for the Eu(III) complex were determined by spectrophotometric UV–vis/pH titrations. Spectra of the solutions at variable pH were collected with a Varian Cary 50 spectrophotometer equipped with an optical fiber probe which was inserted in the titration cell (optical path: 1 cm). The cell containing the ligand (*C*°_L_ = 0.045 mM) and an equimolar quantity of Eu(III) was titrated with NaOH and the pH corresponding to each spectrum was measured as described above. Formation constants were calculated by fitting the absorbance values at various wavelengths by using HypSpec^39^ program.

### DFT Calculations

The paramagnetic Eu(III) ion has been replaced by Y(III) which is a suitable substitute.^8^ Geometry optimizations were carried out at DFT level in PCM^40^ water using the B3LYP^41,42^ exchange–correlation functional. The 6-31+G(d) basis set was used for the ligand atoms, while Y(III) ion was described by the quasi-relativistic small core Stuttgart-Dresden pseudopotential and the relative basis set.^43^ All the final structures were checked to be minima by vibrational analysis. ESP fitting charges were calculated by using the CHelpG scheme.^44^ All calculations were carried out with Gaussian16.^45^

### Fluorometric Titrations

In the titration of the complexes, progressive amounts of BSA (up to 180 μM for [Eu(b*iso*Qcd)(H_2_O)_2_]OTf and up to 24 μM for [Eu(bpcd)(H_2_O)_2_]Cl]) were added to solutions containing the complexes (80 μM). Physiological solutions were prepared MOPS buffered (pH = 7.4) and as isotonic systems (0.9% w/v NaCl). After each addition of BSA, UV–vis, fluorescence, and excitation spectra as well as the Eu(III)-^5^D_0_ excited-state lifetimes were recorded at 298 K.

Titration of BSA was carried out at 298 K. Progressive amounts of [Eu(b*iso*Qcd)(H_2_O)_2_]OTf or [Eu(bpcd)(H_2_O)_2_]Cl up to 200 μM were added to MOPS-buffered physiological solution containing 5 μM of BSA. Integrated areas of BSA fluorescence were analyzed using the MS-Excel cEST macro.^46^ Model robustness was checked by statistical tests (Akaike information criterion) implemented in the associated tool Solverstat.^47^

Competitive fluorimetric titration experiments were carried out involving warfarin (up to 20 μM, in the case of [Eu(bpcd)(H_2_O)_2_]Cl and up to 100 μM in the case of [Eu(b*iso*Qcd)(H_2_O)_2_]OTf) in a MOPS-buffered solution containing BSA-complex adducts (molar ratio [Eu(bpcd)(H_2_O)_2_]Cl: BSA = 4:1; [Eu(bpcd)(H_2_O)_2_]Cl = 80 μM. Molar ratio [Eu(b*iso*Qcd)(H_2_O)_2_]OTf: BSA = 1:1; [Eu(b*iso*Qcd)(H_2_O)_2_]OTf = 80 μM). After each addition of warfarin, Eu(III) luminescence emission spectrum was recorded. Similar experiments were carried out with ibuprofen (up to 0.4 mM) and digitoxin (up to 1 mM).

### Isothermal Titration Calorimetry

Titrations were performed using a TA Instruments TAMIII thermostat equipped with a nanocalorimeter operating at *T* = 298.15 K and with a stirring rate of 50 rpm. The sample cell was filled with a solution (*V* = 0.7 mL) of 0.25 mM of BSA in MOPS buffer (pH 7.4). Reference cell was filled with MOPS buffer. The titration syringe contained a solution of the complex (1.5–3.0 mM) in MOPS buffer. In the case of the [Eu(bisoQcd)(H_2_O)_2_]Cl complex, it was necessary to add 10% v/v EtOH, due to the low solubility in pure water.

The heats of dilution were estimated by using identical injections of buffer solution into the protein. All calorimetric data were corrected with the heat of dilution by subtracting the blank from the experiments. At least two independent titration experiments were performed to confirm consistency. Data analysis was performed using HypDeltaH.^39^

### Molecular Docking

The Y(III) analogues of the two Eu(III) complexes depicted in Figure 1 were docked against the bovine serum albumin crystal structure (PDB code: 4F5S) using the Autodock suite ver. 4.2.6.^48^ Two flexible docking experiments for each complex against two binding sites were performed. These were chosen in order to include the two tryptophan residues of the structure. The flexible residues were selected according to a cutoff of 6 Å for each tryptophan residue: R194, L197, R198, S201, W213, N217, A341, V342, S343, D450, L454 and E16, E17, F126, K127, A128, D129, E130, K132, F133, W134, N158, N161, Q165 around W213 and W134, respectively. Since Autodock suite does not include by default the Y(III) parameters in its force-field, those were manually added to the parameter’s library. For each Autodock run, a cluster analysis over 100 binding poses were performed.

### Molecular Dynamics Simulations

From each docking cluster analysis, a docked structure from the better cluster was chosen to perform small molecular dynamics simulations. All MD simulations were carried out using the GROMACS program, ver. 2016.5.^49^ Since the Y(III) parameters are not included by default in the most commonly used force-fields, they had to be included manually into the force-field; Y(III) was treated as an ion. Therefore, the parameters of each component of the docked structure was prepared differently. The topology of the ligand molecule without Y(III) was prepared using the ANTECHAMER suite,^50^ and the charges from the DFT calculations were included manually. The protein was parametrized using the pdb2gmx from GROMACS with the AMBER99SB force-field. Y(III) parameters were included manually in the force-field.^51^ Water molecules and ions (0.154 M of Na^+^/Cl^–^ to mimic physiological conditions) were added to complete the system. The system was then equilibrated through a complete workflow: steepest descents minimization of 5000 steps, NVT equilibration of 100 ps, NPT equilibration of 100 ps, and MD production under the NPT ensemble for 100 ns at room temperature. A pull code with 5000 kJ mol^–1^ nm^–2^ of force between the nitrogen, oxygen, and Y(III) atoms was included in the MD production to keep the integrity of the complexes. All calculations were performed within a GPU node available by the computational platform from the “Centro Piattaforme Tecnologiche” of the University of Verona.

## Results and Discussion

### Ligand Protonation and Complex Formation

The best fit protonation constants for the b*iso*Qcd ligand (Figure S4) show the presence of four protonated species, as previously found for the bpcd counterpart and the quinoline-containing analogue bQcd (Table 1).^8,35^ The log*K* values for the b*iso*Qcd ligand suggest the presence of two moderately strong acidic sites and two weakly acidic ones. The first protonation constant (log*K*_1_ = 9.22) can be assigned to a tertiary amine, in agreement with those already reported.^52^ The spectrophotometric acid–base titration (Figure S5) shows that molar absorbance is almost constant in the 10.5–8.5 pH range and increases at lower pH with the formation of bi- and triprotonated species. This indicates that both isoquinoline moieties are protonated.^53^ The last species can be associated with acetate protonation.^54^

**Table 1:** Protonation Constants (log*K*_j_) for the b*iso*Qcd Ligand and Complex Formation Constants (logβ_j_)^a^

equilibrium	b*iso*Qcd	bpcd^35^	bQcd^8^	CDTA^55^
L+H⇆HL	9.27 ± 0.03	9.72	9.37	9.43
HL+H⇆H_2_L	5.86 ± 0.07	5.87	5.85	6.01
H_2_L + H⇆H_3_L	3.43 ± 0.07	2.94	3.46	3.68
H_3_L + H⇆H_4_L	1.62 ± 0.09	2.22	1.79	2.51
L+Eu⇆EuL	10.53 ± 0.04	11.19	9.97	19.6
L + Eu ⇆EuL(OH) + H	-	2.18	-	-

a with Eu(III) at *T* = 298.2 K and μ = 0.1 M NaCl. Data for bpcd, bQcd (*N,N′*-bis(2-quinolinmethyl)-trans-1,2-diaminocyclohexane *N,N′*-diacetate), and CDTA (1,2-diaminocyclohexane-*N,N,N′,N*′-tetraacetic acid) are also reported. Charges omitted for clarity.

**Figure 3:**
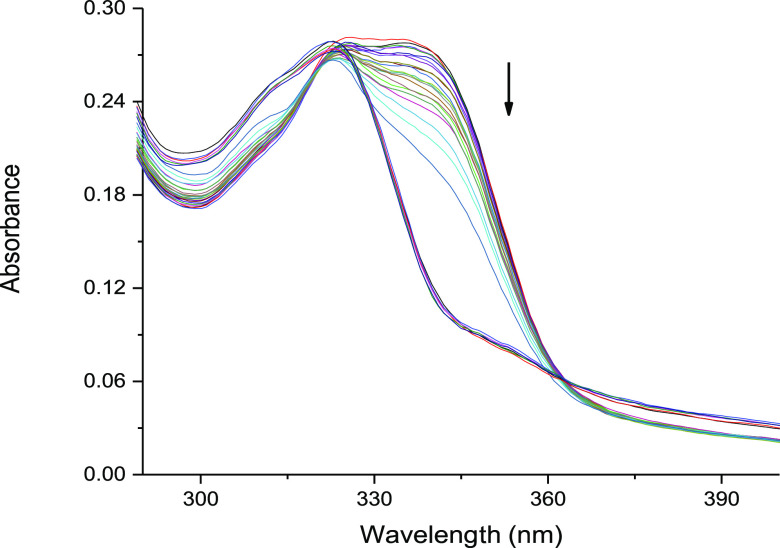
UV–vis absorption spectra changes during the acid–base titration (pH 2.4–11.7) of the ligand b*iso*Qcd (0.045 mM) in the presence of equimolar Eu(III).

**Figure 4:**
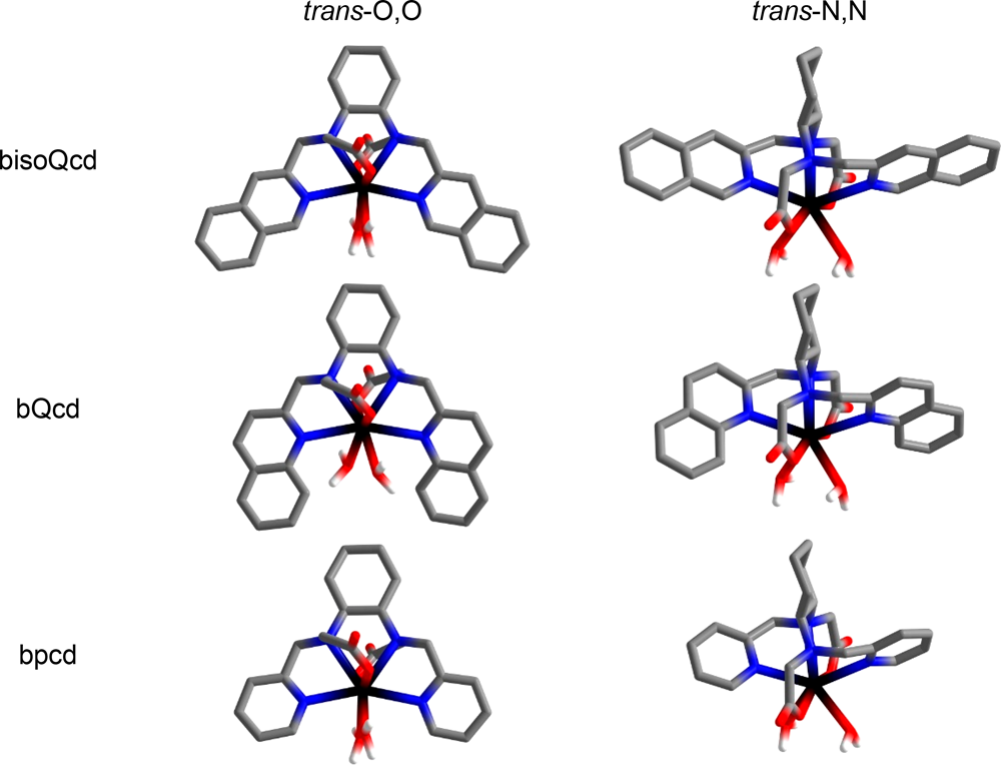
Minimum energy structures of the [Y(L)(H_2_O)_2_]^+^ complexes (L = b*iso*Qcd, bQcd, bpcd). Hydrogens bonded to the C atoms are omitted for clarity.

The analysis of spectrophotometric data (Figures 3 and S6) provides a speciation model with only a 1:1 metal–ligand complex formed ([Eu(b*iso*Qcd)(H_2_O)_2_]^+^). Based on the obtained complex formation constants, at physiological pH = 7.4 the 1:1 species is largely dominant (>99% calculated for *C*_Eu_ = *C*_bisoQcd_ = 10 μM, using the constants in Table 1). The obtained stability is comparable to that found previously for other complexes (Table 1). The distinct binding strength of the three moieties and the steric hindrance may account for the observed slight differences. The higher stability of the [Eu(b*iso*Qcd)(H_2_O)_2_]^+^ complex with respect to the bQcd derivative is ascribable to the higher basicity of isoquinoline compared to quinoline.^53^ This can be deduced from the comparison of the energies of the Yttrium counterparts, the possible isomers^8^ [Y(b*iso*Qcd)(H_2_O)_2_]^+^ and [Y(bQcd)(H_2_O)_2_]^+^ shown in Figure 4. The isomers with b*iso*Qcd are more stable than the corresponding ones involving the bQcd ligand by 6.8 and 8.9 kcal mol^–1^, respectively. This difference seems related with the metal-N_hetero_ bonds which are clearly shorter in [Y(b*iso*Qcd)(H_2_O)_2_]^+^ (0.14 and 0.08 Å for the *trans*-*O,O* and *trans*-*N,N* isomers, respectively) and similar to those found in the (somewhat more stable) complex with bpcd (Table S1). Also, in both cases, the *trans*-*O,O* isomer is the most stable (Δ*G*(*trans*-O,O-*trans*-N,N) = −3.0 and −5.1 kcal mol^–1^ for the complex with bisoQcd and bQcd, respectively), suggesting it as the prevalent form in solution.

### Luminescence

**Figure 5:**
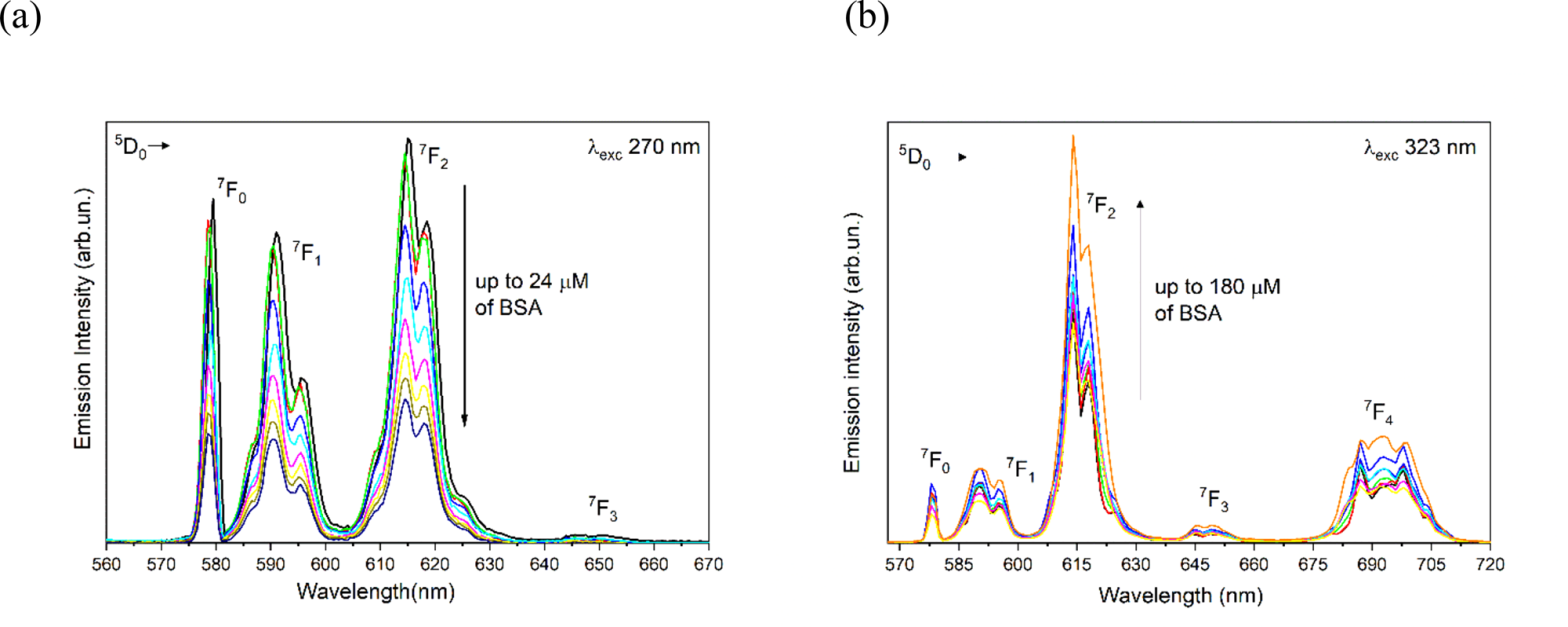
Evolution of the Eu(III) luminescence emission of (a) [Eu(bpcd)(H_2_O)_2_]Cl complex (80 μM) upon addition of BSA in the 0–24 μM concentration range and (b) [Eu(b*iso*Qcd)(H_2_O)_2_]OTf complex (80 μM) upon addition of BSA in the 0–180 μM concentration range, at 298 K.

Upon titration of the Eu(III) complexes with increasing amount of BSA, we observed two opposite trends. In the case of [Eu(bpcd)(H_2_O)_2_]Cl, a gradual decrease of the Eu(III) luminescence intensity was detected (Figure 5a). Conversely, an enhancement of the lanthanide emission was observed for the complex [Eu(b*iso*Qcd)(H_2_O)_2_]OTf (Figure 5b).

The spectral fingerprint of the hypersensitive ^5^D_0_ → ^7^F_4_ transition is particularly affected by the addition of BSA. This behavior suggests a change in the nature of the primary donors to the Eu(III) center,^56^ as the oxygen atom of a water molecule can be displaced by a functional group of the protein, as discussed later in the paper.

Based on the overlap between the electronic spectra of BSA and the [Eu(bpcd)(H_2_O)_2_]Cl complex in the range 260–290 nm, luminescence spectra of the latter cannot be recorded upon excitation in the indicated wavelength domain at a BSA concentration >40 μM (final experimental BSA–complex molar ratio of around 1:3). This is a *conditio sine qua non* for respecting the limits of validity of the Lambert–Beer law (Abs over 3 units). On the contrary, the concentration limit of BSA was extended to 180 μM (2.25:1 BSA–complex molar ratio) for the [Eu(b*iso*Qcd)(H_2_O)_2_]OTf derivative.

**Figure 6:**
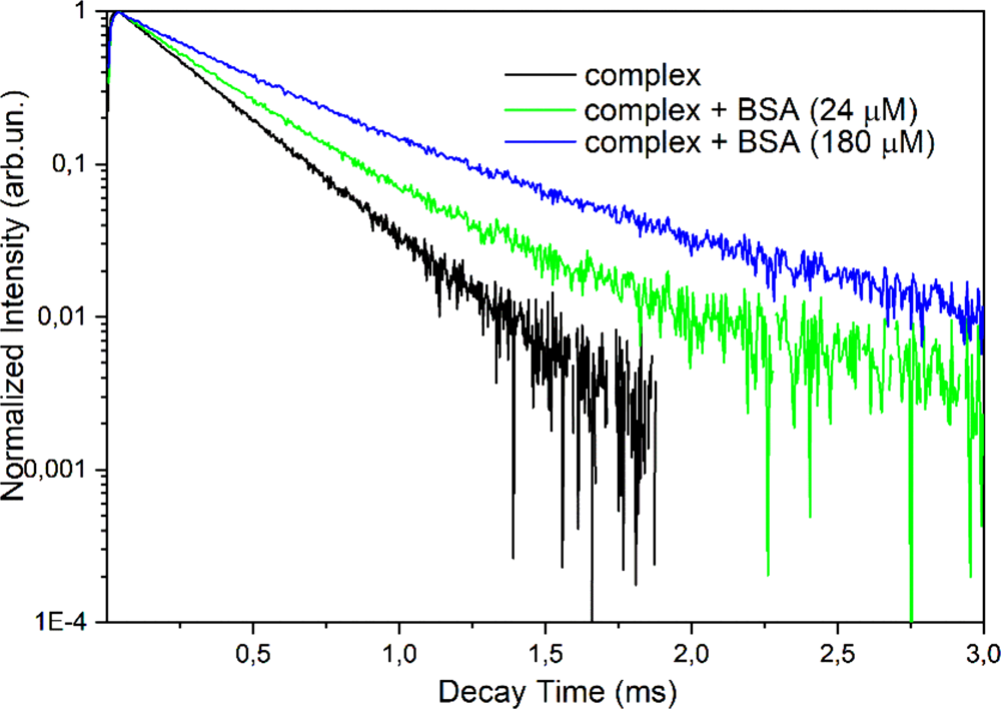
Luminescence decay curves of the ^5^D_0_ excited state of Eu(III) for the complex [Eu(b*iso*Qcd)(H_2_O)_2_]OTf complex (80 μM) upon addition of BSA.

To gain further insights into the interaction, the luminescence decay of the ^5^D_0_ excited state was recorded. Figure 6 shows the collected profiles for the complex involving b*iso*Qcd as a ligand at different protein concentrations. Under the same experimental conditions, the compound [Eu(bpcd)(H_2_O)_2_]Cl showed no significant change in the observed lifetime (ca. 0.30 ms).

The blue and green decay curves cannot be fitted by a single exponential function when BSA and the complex are both present in solution, likely due to the presence of more than one emitting species (complex/protein adducts) in solution. In such cases, the values of the 1/e folding time is given instead of the observed lifetime. The increase of the ^5^D_0_ excited state lifetime upon addition of the protein [0.28 ms for the free complex, 0.34 and 0.50 ms after the addition of BSA (24 μM and 180 μM, respectively)] can be attributed to a lower efficiency of the multiphonon relaxation process (i.e., process able to quench the excited state of the lanthanide ion in a nonradiative way).^57,58^ In fact, in the binding site some functional groups of the protein may replace water molecules in the inner coordination sphere of the metal ion. In this regard, literature data confirm that Ln(III) complexes wherein the metal center is coordinated by two water molecules (as in our case) are susceptible to ligand displacement by competitive binding to endogenous serum anions, such as carbonate, or protein carboxylic acid residues.^59^ Based on these considerations, the Eu(III) luminescence intensity increases, as well as the quantum yield. On the contrary, the protein/complex interaction seems not to affect the number of water molecules bound to the metal ion in the [Eu(bpcd)(H_2_O)_2_]Cl complex. The decrease of the Eu(III) emission intensity could be in part due to the Inner Filter Effect (IFE).^60^ IFE is an alternative signal transduction mechanism that relies on the reduction of a fluorophore’s emission, caused by preventing its excitation by absorbing light at its excitation wavelength by other chromophores: in the case of [Eu(bpcd)(H_2_O)_2_]Cl complex, the excitation wavelength of the complex (around 270 nm) is partially absorbed by BSA, which presents an absorption maximun around 280 nm. On the other hand, the aforementioned decrease of the Eu(III) emission intensity could be also connected to a less efficient ligand-to-metal energy transfer. In this regard, generally the interaction with BSA involves the tryptophan residues in domains I and II (Trp-134 and Trp-213; Figure S3),^61^ which could bind the metal ion instead of the antenna. Despite this observation, the excitation spectrum of [Eu(bpcd)(H_2_O)_2_]Cl shows the presence of a peak around 265 nm which undergoes a blue-shift toward 262 nm upon addition of the protein (Figure S7a). This peak is indicative of the luminescence sensitization of Eu(III) by pyridine rings; thus, we can rule out the involvement of the tryptophan rings in the sensitization mechanism, as their typical excitation peak around 280 nm is absent. Instead, the aforementioned blue-shift could be due to a change of the energy of the electronic levels of the pyridine antenna, thus being detrimental to the efficiency of the energy transfer.

The unusual behavior of the [Eu(bpcd)(H_2_O)_2_]Cl complex points out a different nature of the protein/complex interaction, which should primarily involve the organic ligand rather than the metal ion. *Vice versa,* the involvement of the Eu(III) coordination sphere of the [Eu(b*iso*Qcd)(H_2_O)_2_]^+^ complex in the interaction with the protein was also confirmed by the change in the asymmetry ratio value^62^
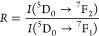
indicative of the degree of asymmetry of the coordination polyhedron around the Eu(III) ion, which passes from 3.80 at the beginning of the titration to 4.20 at the end. In agreement with previous considerations, the *R* value remains basically constant (around 1.55) during BSA additions for the [Eu(bpcd)(H_2_O)_2_]Cl complex. Moreover, the recorded excitation spectra for [Eu(b*iso*Qcd)(H_2_O)_2_]OTf (Figure S7b) reveal no or negligible energy transfer mechanisms involving chromophoric protein groups (i.e., no excitation peak around 280 nm in the spectrum).

**Figure 7:**
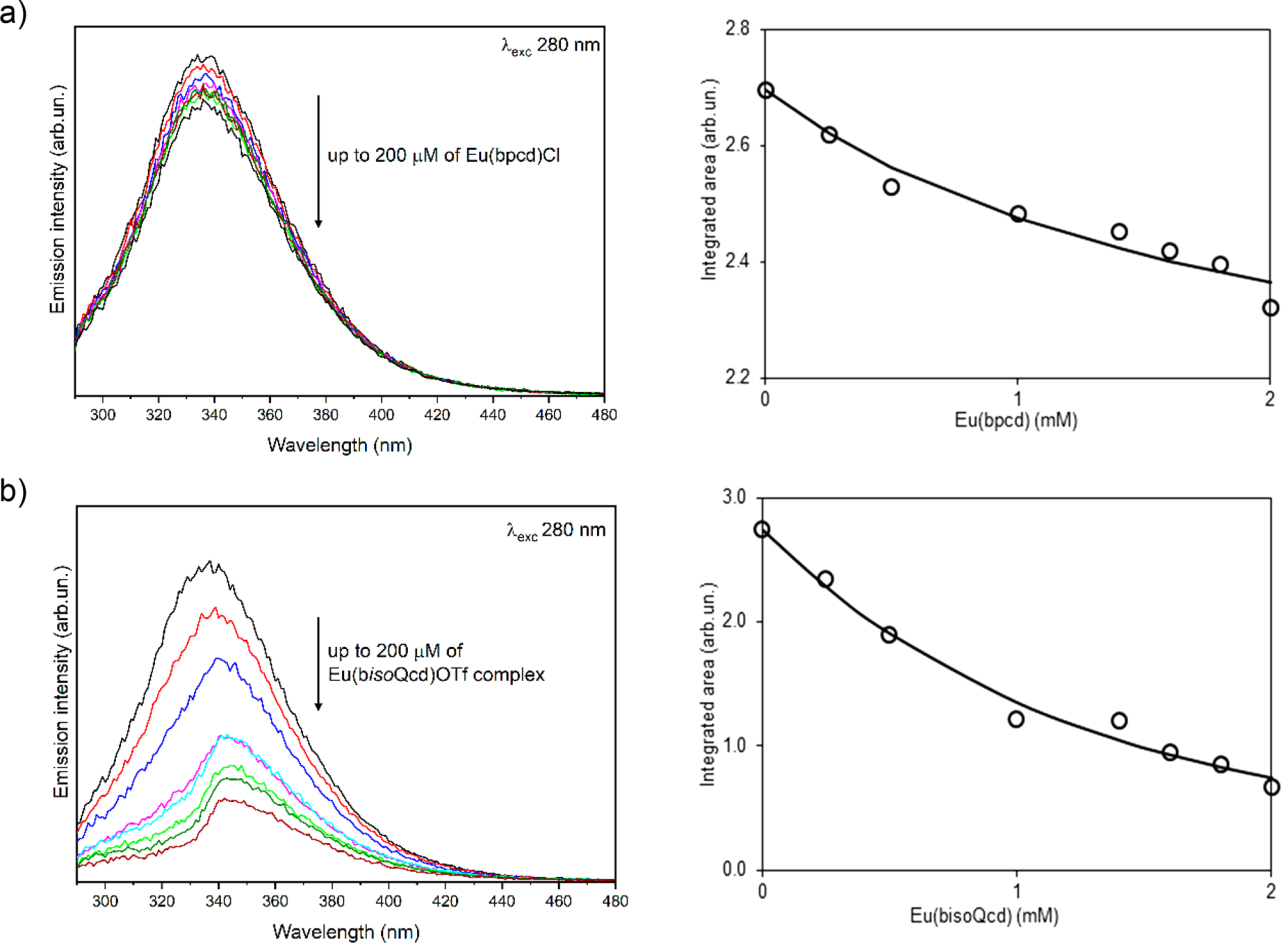
Evolution of the fluorescence spectrum of BSA (5 μM solution) upon addition of (a) [Eu(bpcd)(H_2_O)_2_]Cl and (b) [Eu(b*iso*Qcd)(H_2_O)_2_]OTf. On the right, the integrated area of each spectrum (○) vs total complex concentration together with the fit (line) obtained with the formation constants in Table 2.

In order to gain more insights into the protein/complex interaction mechanism, we also investigated the evolution of the protein fluorescence upon addition of the Eu(III) complex. In this case, the limits of validity of the Lambert–Beer law cover both a wider complex concentration range (up to 200 μM) and a larger complex/protein molar ratio (up to 40:1). In both cases, we observed a decrease of the fluorescence intensity, which is particularly clear for the derivative with b*iso*Qcd as a ligand (Figure 7).

In addition, the maximum emission peak of the protein (around 335 nm) undergoes a 10 nm red-shift. Therefore, the corresponding transition (π* → π) occurs in a locally more polar medium. In other words, the BSA binding to the [Eu(b*iso*Qcd)(H_2_O)_2_]OTf complex leads to a protein conformational change, in turn decreasing the rigidity of the Trp environment and/or exposing such an amino acid residue to a more hydrophilic environment.^63^ Since upon excitation around 280 nm we mainly excite Trp side chains and to a lesser extent the tyrosine residues,^33^ the significant change of the fluorescence intensity of the protein is indicative of an interaction taking place close to the Trp residues discussed above. On the contrary, the small change of fluorescence intensity detected for [Eu(bpcd)(H_2_O)_2_]^+^ predicts an interaction located in a different protein site.

The fluorescence decrease by our complexes can result from a variety of phenomena, such as collisional (or dynamic) quenching and static quenching.^64^ Regarding the former (a bimolecular process), upon contact of BSA with some molecule in solution (e.g., the Eu(III) complex) via an inelastic collision, the fluorophore reaches its excited state, then deactivated by heat release. Conversely, in the static case the fluorophore can form nonfluorescent adducts with the quencher. In order to discriminate between static or dynamic quenching, we measured the lifetime of the protein excited state. The fluorescence decay of BSA was best fitted with a biexponential function, and the corresponding averaged lifetimes, ⟨τ_av_⟩, remained unaltered upon addition of both complexes (around 7.5 ns). This indicated that the quenching of the fluorescence follows a static mechanism and a ground-state complex between BSA and each Eu(III) complex should be present in solution.^64^

The fluorescence data were analyzed by the well-known Stern–Volmer plot.^65^ As shown in Figure S8, a linear correlation of the fluorescence intensities *F*_0_/*F* with the concentration is undoubtedly observed only in the case of [Eu(bpcd)(H_2_O)_2_]Cl complex. The determined Stern–Volmer constant *K*_sv_ of this compound is 2089 M^–1^. The analysis of BSA emission data, by means of the program MS-Excel cEST macro, provided best-fitting models (Figure 7, right) which correspond to the formation of a 1:1 adduct for the [Eu(bpcd)(H_2_O)_2_]Cl complex (Figure 7a) and of 1:1 + 1:2 species for the original complex [Eu(bisoQcd)(H_2_O)_2_]^+^ (Figure 7b). The obtained adduct formation equilibrium constants (log*K*) are reported in Table 2. Interestingly, the Log*K*_sv_ value reported above for the [Eu(bpcd)(H_2_O)_2_]Cl complex is in good agreement with the formation constants for the complex/BSA adduct reported in Table 2 (Log*K*_sv_ = 3.32 ± 0.02; Log*K* = 3.7 ± 0.6). As expected for the static quenching regime,^64^ the Stern–Volmer quenching constant is identified with the adduct association constant.

**Table 2:** Formation Constants for the Complex/BSA Adducts Obtained from Fluorimetric and ITC Titrations

L =	reaction	log*K* Fluorimetry	log*K* ITC	Δ*H* (kJ mol^–1^)
bpcd	BSA + EuL ⇋ BSA[EuL]	3.7 ± 0.6	3.61 ± 0.07	–13.1 ± 0.1
bisoQcd	BSA + EuL ⇋ BSA[EuL]	3.9 ± 0.2	-	–16 ± 3
	BSA[EuL] + EuL ⇋ BSA[EuL]_2_	3.6 ± 0.4	-	–32 ± 4

### Isothermal Titration Calorimetry

**Figure 8:**
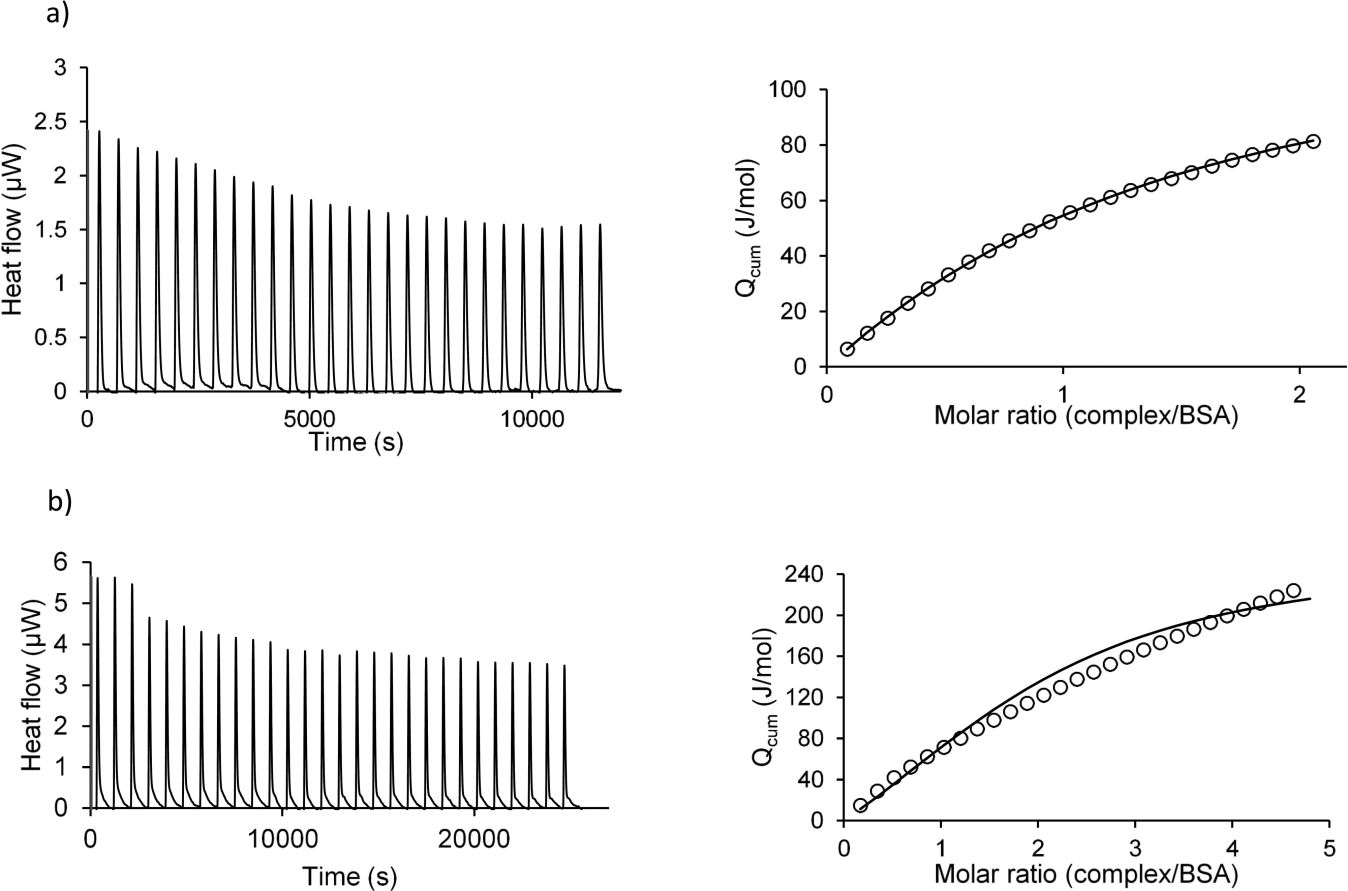
Calorimetric titrations of (a) BSA (0.25 mM) with [Eu(bpcd)(H_2_O)_2_]Cl (1.5 mM). Solvent: aqueous solution of MOPS, 13 mM; pH = 7.4. Final Eu/BSA molar ratio = 2.14. (b) BSA (0.25 mM) with [Eu(bisoQcd)(H_2_O)_2_]OTf (3 mM). Solvent: aqueous solution of MOPS 13 mM with EtOH 10% v/v; pH = 7.4. Final Eu/BSA molar ratio: 4.8. On the right, the experimental (○) and calculated (line) *Q*_cum_ (cumulative heat exchanged/total moles of added reactant) vs complex/BSA molar ratio.

As initially different models provided a reasonable fit of the fluorimetric data and these data could be affected by the inner filter effect (IFE), the BSA–complex interaction was also studied by a different experimental technique such as isothermal titration calorimetry (ITC). For the [Eu(bpcd)(H_2_O)_2_]Cl (Figure 8a) a 1:1 model well fits the heat data, resulting in log*K* = 3.61 and a negative formation enthalpy (Δ*H*) (Table 2). This value of log*K* agrees with the constant obtained from luminescence data (Figure 7a), where a small change in the intensity of the protein fluorescence was observed upon titration with the complex. This result suggests that IFE does not affect dramatically the protein fluorescence in the presence of [Eu(bpcd)(H_2_O)_2_]Cl. For the [Eu(bisoQcd)(H_2_O)_2_]^+^ complex, ITC data (Figure 8b) support the presence of 1:1 + 1:2 protein–complex adducts, established by a stronger interaction compared to the other derivative. When both log*K* and Δ*H* were set as free parameters in ITC data fitting, very large errors were obtained; hence, the values are not reported in Table 2.

### Ligand Competition Studies

To identify the BSA sites involved in the interaction with the Eu(III) complexes, fluorimetric titrations using site-selective competitive ligands (the clinically established drugs ibuprofen, warfarin, and digitoxin)^66−68^ were carried out. Warfarin is selective for the external site (domain I) where Trp-134 residue is present, while ibuprofen for the domain II, associated with the inner Trp-213 residue (Figure S2). Digitoxin shows a selective interaction with the domain III, not fluorescent, and with which our complexes seem not to interact.^69^

**Figure 9:**
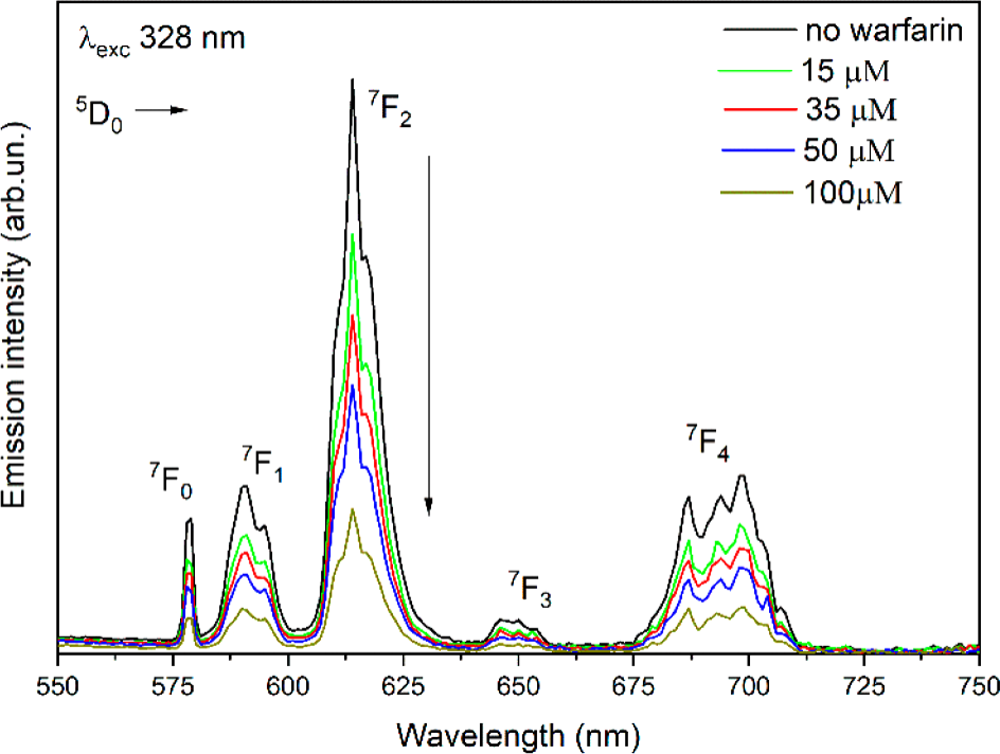
Luminescence of the complex [Eu(b*iso*Qcd)(H_2_O)_2_](OTf) (80 μM) interacting with BSA (80 μM) upon titration with warfarin (up to 100 μM); room temperature; solvent: aqueous solution of MOPS, 13 mM; pH = 7.4.

Upon addition of warfarin to the solution containing the BSA/[Eu(bpcd)(H_2_O)_2_]Cl adduct, a decrease of the Eu(III) luminescence intensity within the experimental error was detected. Since the BSA–warfarin adduct is associated with a log*K*_1_ = 5.07,^70^ higher than the one calculated for the BSA/[Eu(bpcd)(H_2_O)_2_]^+^, there is no competition between warfarin and such a complex for the domain I. Likewise, the [Eu(bpcd)(H_2_O)_2_]Cl complex does not interact with the domain II, as the Eu(III) luminescence intensity does not change upon addition of ibuprofen to the BSA/complex adduct. On the contrary, the addition of warfarin to the BSA adduct(s) with [Eu(bisoQcd)(H_2_O)] resulted in a significant decrease (around 85%) of the Eu(III) luminescence intensity (Figure 9).

This behavior, opposite the one highlighted in Figure 5b, points out the Eu(III) complex replacement by warfarin at site I (Trp 134). However, both fluorimetric and calorimetric titrations suggest that [Eu(bisoQcd)(H_2_O)_2_]OTf can interact with BSA in an additional position. Since the Eu(III) luminescence intensity does not change for this compound upon addition of both ibuprofen and digitoxin, the second interaction site is different from those of the two here studied drugs.

In the case of warfarin and ibuprofen, it was checked that each drug interacts with the protein by monitoring, upon titration, the BSA fluorescence stemming from the adduct with the complexes. The addition of these drugs results in a significant decrease of the protein fluorescence intensity. In order to better understand the nature of the protein/complex interactions, molecular docking and molecular dynamics simulations were performed.

### [Y(bpcd)(H_2_O)_2_]^+^ Complex

**Figure 10:**
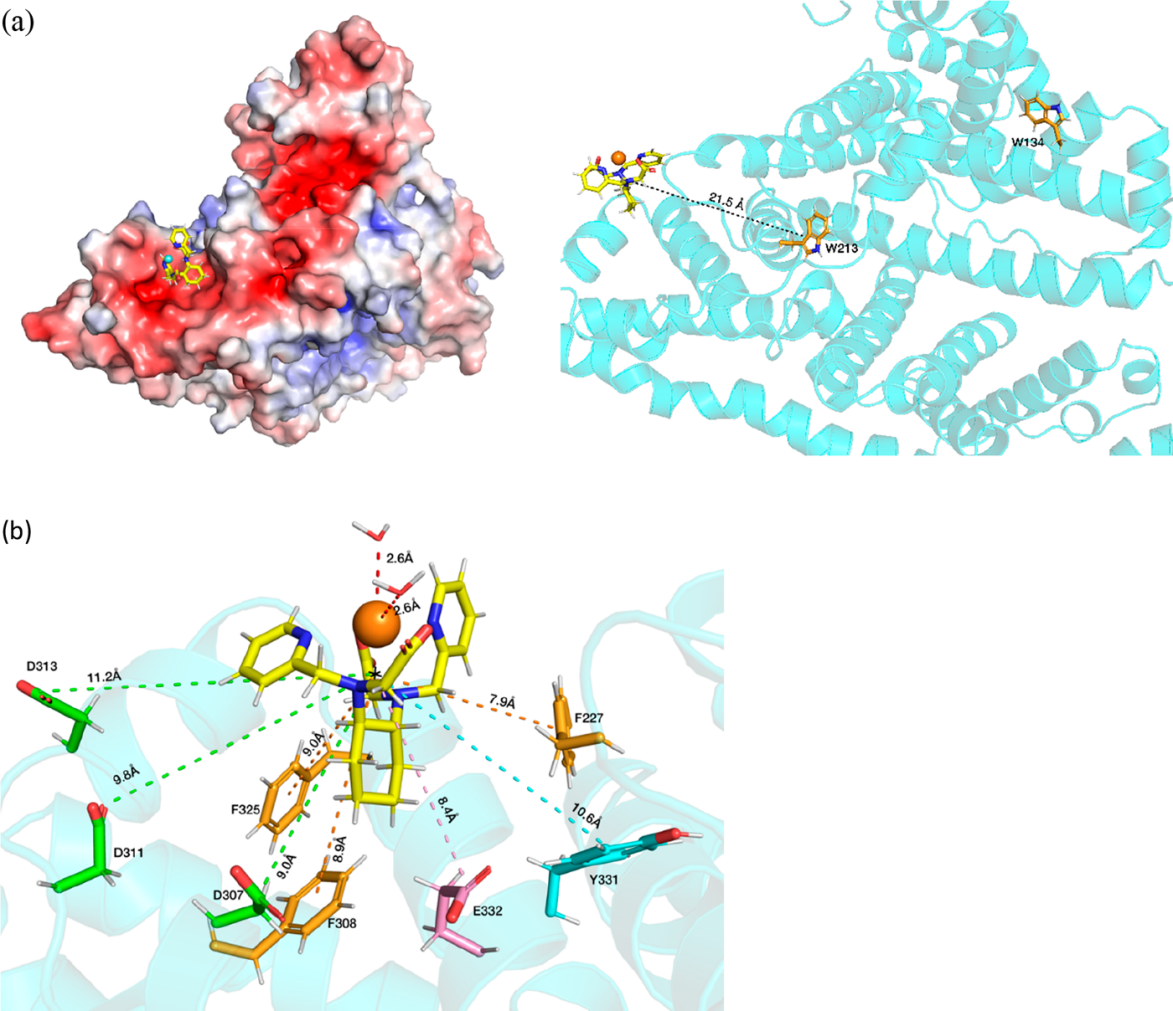
(a) BSA superficial interaction site with the [Y(bpcd)(H_2_O)_2_]^+^ complex. The electrostatic potential is represented (electronegative cavities are red colored) (left) as well as the distance between the interacting complex and the buried Trp 213 (right). (b) Close-up on the [Y(bpcd) (H_2_O)_2_]^+^ that lies on the superficial cavity and representation of the amino acid residues surrounding the complex (distances averaged along the simulation).

When the computationally equivalent [Y(bpcd)(H_2_O)_2_]^+^ complex is docked close to the sites I and II (associated with the presence of Trp134 and Trp213, respectively), it does not remain in these binding sites. It explores all the protein surfaces after the first nanoseconds of simulation. Then, [Y(bpcd)(H_2_O)_2_]^+^ interacts with a superficial cavity where it spends half the simulation time (50 ns) (Figures 10 and S9).

The amino acid residues located at the external surface of this cavity and therefore likely involved in the interaction with the pyridine-based complex consist of several groups arising from four residues of phenylalanine (F227, F308, F325, and F329) and two residues of tyrosine (Y318 and Y331). In this superficial area also, five negatively charged carboxylic groups are present [from three aspartic acids (D307, D311, D313) and two glutamic acids (E320, E332); Figure10b]. This piece of evidence is in line with the possible interactions that [Y(bpcd)(H_2_O)_2_]^+^ can establish with the protein. In particular, the positive charge of the complex can give rise to a Coulombic interaction with the negatively charged aspartic and glutamic acid side chains. The pyridine rings of the ligand can interact by means of van der Waals contacts with the side groups of aromatic amino acids listed above. As mentioned in the Introduction, since such amino acids (phenylalanine and tyrosine) contribute to the protein fluorescence to a small extent, their involvement in the interaction with the complex [Eu(bpcd)(H_2_O)_2_]^+^ could affect only marginally the fluorescence spectrum of BSA (Figure 7a).

It is worth noting that upon interaction the metal ion does not lose the bound water molecules (two), as shown in the Figure S10a, where the graphical representation of the radial distribution function is reported, as a function of the O(water)–Y distance. In fact, the number of water molecules in the inner coordination sphere of the metal ion (at 2.6 Å) is around two, both in the simulation in bulk water and upon interaction with the protein. This is in good agreement with the analysis of the Eu(III) luminescence decay curves discussed in the previous section.

### [Y(bisoQcd)(H_2_O)_2_]^+^ Complex

[Y(b*iso*Qcd)(H_2_O)_2_]^+^ shows a selective interaction with the outer binding site containing the Trp residue 134, as it remains in this protein superficial area through all the simulation time (100 ns). On the contrary, no interaction occurs with the buried Trp213, as in the case of the [Y(bpcd)(H_2_O)_2_]^+^ derivative. From the thermodynamic point of view, based on the binding energy analysis obtained from docking (Figure S11), this inner site is not suitable for both studied complexes.

If we focus on the superficial binding site, the distance between Y(III) and Trp134 was constant (around 6.8 Å) over all simulation time (Figure S12). In particular, we noticed the presence of a “non-conventional” hydrogen bond^71^ involving the aromatic ring of Trp 134 as H-bond acceptor and a C–H bond of the isoquinoline ring as donor (Figure S13). The phenomenon of the “CH/π interaction” in organic and biological chemistry has been explored and reviewed by Nishio et al. in a very interesting way.^72^

**Figure 11:**
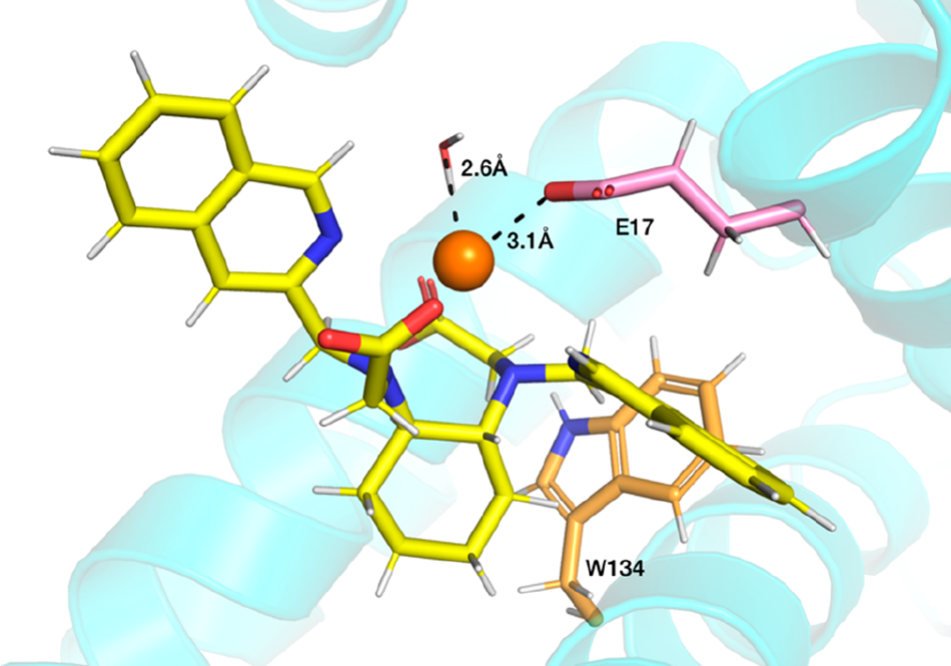
Snapshot of the interaction site of BSA with the [Y(b*iso*Qcd)(H_2_O)]^+^ complex.

These results are in perfect agreement with the outcomes associated with the analysis of the BSA fluorescence upon interaction with the complex and the competitive titration experiments with the drug warfarin. In addition, one coordinated water molecule is displaced by one monodentate carboxylic group of the glutamic acid residue 17 (E17) (Figure 11).

In addition, this finding is in agreement with the increase of the observed luminescence lifetime of Eu(III) during the titration of [Eu(bisoQcd)(H_2_O)_2_]^+^ complex with the protein (Figure 6). In fact, the removal of one water molecule from the inner coordination sphere of Eu(III) reduces the multiphonon relaxation process. A radial distribution analysis (Figure S10b) confirmed that, while in bulk water the metal ion has two water molecules in its coordination sphere (at a distance of ∼2.5 Å), when the complex is docked into the binding site close to Trp134, the ion just retains one water molecule. Interestingly, an inspection of the Y coordination environment for both complexes revealed that the bond distances between the metal ion and the donor atoms of the ligands are in practice unaffected by the interaction with protein (Figures S14 and S15). Focusing on the complex geometry, only small differences in the orientation of the heteroaromatic fragments are noticed for [Y(b*iso*Qcd)(H_2_O)]^+^ upon interaction with BSA (Figure S16). In contrast, a significant change in the orientation of the pyridine rings was detected in the case of the [Y(bpcd)(H_2_O)_2_] ^+^ complex. In this case, the lone pair of one pyridine nitrogen is not pointing precisely toward the metal ion (see Figure S17 for details).

To sum up, molecular docking calculations led to results in agreement with those achieved via spectroscopy. In fact, on one hand the interaction of the [Y(b*iso*Qcd)(H_2_O)_2_]^+^ complex with the protein mainly affects the environment of the metal ion (i.e., displacement of a water molecule by E17, acting as a monodentate *O*-donor ligand). On the other hand, the coordination sphere of [Y(bpcd)(H_2_O)_2_]^+^ remains the same before and after the interaction with the metal center playing no key role. The interaction is instead mediated by the ligand scaffold, establishing various interactions with the model protein based on different topological (morphological and chemical) complementarities.

At this stage, it is useful to recall the spin selection rules underlying the energy exchange mechanism.^73^ The efficiency of this process—governing a nonradiative energy transfer and involving the triplet state of a ligand and the excited states of lanthanide ions—is optimal if (i) the energy overlap of the absorption band of the donor (D) and the emission band of the acceptor (A) is high; (ii) the D–A distance is small; and (iii) the orientation of the orbitals involved in the mechanism is such that their overlap is good.^73^ Since the orbitals of the pyridine rings should be involved in such a mechanism, the observed change in their orientation upon protein interaction could worsen the orbital overlap discussed at the point (iii) and, in turn, the efficiency of the exchange mechanism. This conclusion is also supported by spectroscopy: apart from the IFE, the deterioration of the ligand-to-metal energy transfer efficiency could contribute to the decrease of the luminescence emission intensity of the [Eu(bpcd)(H_2_O)_2_]Cl complex during the titration with BSA (Figure 5a).

## Conclusions

The new [Eu(b*iso*Qcd)(H_2_O)_2_]^+^ complex was synthesized, in-depth characterized, and its solution behavior investigated. The stability is slightly higher than that of the previously studied [Eu(bQcd)(H_2_O)_2_]^+^ counterpart, due to the larger basicity of the isoquinoline compared to quinoline. The speciation showed that the EuL complex is the only species present at physiological pH, thus making it a good candidate for luminescent sensing of bioanalytes.

The interaction of [Eu(b*iso*Qcd)(H_2_O)_2_]^+^ and [Eu(bpcd)(H_2_O)_2_]^+^ with BSA, by us conceived as a potential competitor, was studied by ITC and by analysis of the lanthanide and protein emission spectra. Experimental data revealed a markedly different behavior of the two complexes. [Eu(bpcd)(H_2_O)_2_]^+^ can form only a 1:1 adduct on an external pocket of the protein as suggested by MD with no involvement of the specific sites of three drugs explored here via competitive titrations. The luminescence decay remains unchanged when the complex interacts with the protein, thus indicating a mere structural role for the metal center (M). The M–L bonds proved strong enough to keep the pharmacophore-like scaffold in fixed positions, thus pointing to specific amino acid residues. On the contrary, [Eu(b*iso*Qcd)(H_2_O)_2_]^+^ directly involves the Eu(III) ion during the interaction with the protein by coordination to a glutamate side chain (E17) in parallel with the loss of one water molecule in the first coordination sphere, thus increasing the luminescence lifetime. It can establish two types of adduct, namely, with 1:1 and 1:2 protein/complex stoichiometry. At least one occurs in the Trp 134 region (protein domain I), as highlighted by the warfarin displacement.

It is worth highlighting that the indications obtained could be exploited in the development of Gd(III)-based blood-pool magnetic resonance imaging (MRI) contrast agents. Indeed, similarly to the two Eu(III) complexes studied here, Gd(III) analogues should retain at least one water molecule upon interaction with the protein and, at the same time, take advantage of the relaxivity increase due to the adduct formation with the protein.^74^

Finally, this work paves the way to future studies aimed at designing and developing new molecular luminescent chiral probes wherein the nature and the scaffold size of the multidentate ligand as well as the stability of the M–L bonds play a role of paramount importance to enable or reduce the accessibility to the Ln(III) center.

## Supporting Information Available

Listings of structural data of BSA and HSA proteins, potentiometric and acid–base spectrophotometric data, luminescence excitation data, Stern–Volmer plots, radial distribution functions, and molecular docking data (coordination distances and geometries) (PDF)
