# Supplementary material for: Effect of the Heteroaromatic Antenna on the Binding of Chiral Eu(III) Complexes to Bovine Serum Albumin
Source: Inorg Chem. 2020 Aug 10;59(17):12564–77. doi: 10.1021/acs.inorgchem.0c01663 (PMC8009522; doi:10.1021/acs.inorgchem.0c01663)
Supplement: Supplementary file 1 — ic0c01663_si_001.pdf [file ic0c01663_si_001.pdf]

# Effect of the heteroaromatic *antenna* on the binding of chiral Eu(III) complexes to bovine serum albumin (BSA)

*Chiara De Rosa,[1] Andrea Melchior,[2*] Martina Sanadar,[2] Marilena Tolazzi,[2] Alejandro Giorgetti,[3] Rui P. Ribeiro[3], Chiara Nardon,[1] Fabio Piccinelli[1*]*

[1] Luminescent Materials Laboratory, Department of Biotechnology, University of Verona and INSTM - UdR Verona, Strada Le Grazie 15, 37134 Verona, Italy

[2] Laboratory of Chemical Technologies, Polytechnic Department of Engineering and Architecture, University of Udine, via Cotonificio 108, 33100 Udine, Italy

[3]Applied Bioinformatics Laboratory, Department of Biotechnology, University of Verona, Strada Le Grazie 15, 37134 Verona, Italy

* corresponding author's email address: fabio.piccinelli@univr.it and andrea.melchior@uniud.it

## Electronic Supplementary Information

```
BSA           1 DTHKSEIAHRFKDLGEEHFKGLVLIAFSQYLQQCPFDEHVKLVNELTEFAKTCVADESHA
HSA           1 DAHKSEVAHRFKDLGEENFKALVLIAFAQYLQQCPFEDHVKLVNEVTEFAKTCVADESAE
                * **** ********** ** ****** ********  ******* ************

BSA          61 GCEKSLHTLFGDELCKVASLRETYGDMADCCEKQEPERNECFLSHKDDSPDLPKL-KPDP
HSA          61 NCDKSLHTLFGDKLCTVATLRETYGEMADCCAKQEPERNECFLQHKDDNPNLPRLVRPEV
                 * ********* ** ** ****** ***** *********** **** * ** *  *

BSA         120 NTLCDEFKADEKKFWGKYLYEIARRHPYFYAPELLYYANKYNGVFQECCQAEDKGACLLP
HSA         121 DVMCTAFHDNEETFLKKYLYEIARRHPYFYAPELLFFAKRYKAAFTECCQAADKAACLLP
                   *  *   *  *  *******************  *  *   * ***** ** *****

BSA         180 KIETMREKVLTSSARQRLRCASIQKFGERALKAWSVARLSQKFPKAEFVEVTKLVTDLTK
HSA         181 KLDELRDEGKASSAKQRLKCASLQKFGERAFKAWAVARLSQRFPKAEFAEVSKLVTDLTK
                *    *     *** *** *** ******* *** ****** ****** ** ********

BSA         240 VHKECCHGDLLECADDRADLAKYICDNQDTISSKLKECCDKPLLEKSHCIAEVEKDAIPE
HSA         241 VHTECCHGDLLECADDRADLAKYICENQDSISSKLKECCEKPLLEKSHCIAEVENDEMPA
                ** ********************** *** ********* ************** *  *

BSA         300 NLPPLTADFAEDKDVCKNYQEAKDAFLGSFLYEYSRRHPEYAVSVLLRLAKEYEATLEEC
HSA         301 DLPSLAADFVESKDVCKNYAEAKDVFLGMFLYEYARRHPDYSVVLLLRLAKTYETTLEKC
                 ** * *** * ******* **** *** ***** **** * *  ****** ** *** *

BSA         360 CAKDDPHACYSTVFDKLKHLVDEPQNLIKQNCDQFEKLGEYGFQNALIVRYTRKVPQVST
HSA         361 CAAADPHECYAKVFDEFKPLVEEPQNLIKQNCELFEQLGEYKFQNALLVRYTKKVPQVST
                **  *** **  ***  * ** **********  ** **** ***** **** *******

BSA         420 PTLVEVSRSLGKVGTRCCTKPESERMPCTEDYLSLILNRLCVLHEKTPVSEKVTKCCTES
HSA         421 PTLVEVSRNLGKVGSKCCKHPEAKRMPCAEDYLSVVLNQLCVLHEKTPVSDRVTKCCTES
                ******** *****  **  **  **** *****  ** ***********  ********

BSA         480 LVNRRPCFSALTPDETYVPKAFDEKLFTFHADICTLPDTEKQIKKQTALVELLKHKPKAT
HSA         481 LVNRRPCFSALEVDETYVPKEFNAETFTFHADICTLSEKERQIKKQTALVELVKHKPKAT
                ***********  ******* *    **********   * *********** *******

BSA         540 EEQLKTVMENFVAFVDKCCAADDKEACFAVEGPKLVVSTQTAL
HSA         541 KEQLKAVMDDFAAFVEKCCKADDKETCFAEEGKKLVAASQAAL
                 **** **  * *** *** ***** *** ** ***   * **
```

**Figure S1.** Comparative sequence analysis of BSA and HSA performed by the free service available at http://web.expasy.org/sim/, using sequence data deposited in the protein data bank (1AO6.pdb and 3V03.pdb for HSA and BSA, respectively). The tryptophan residues are yellow-highlighted.

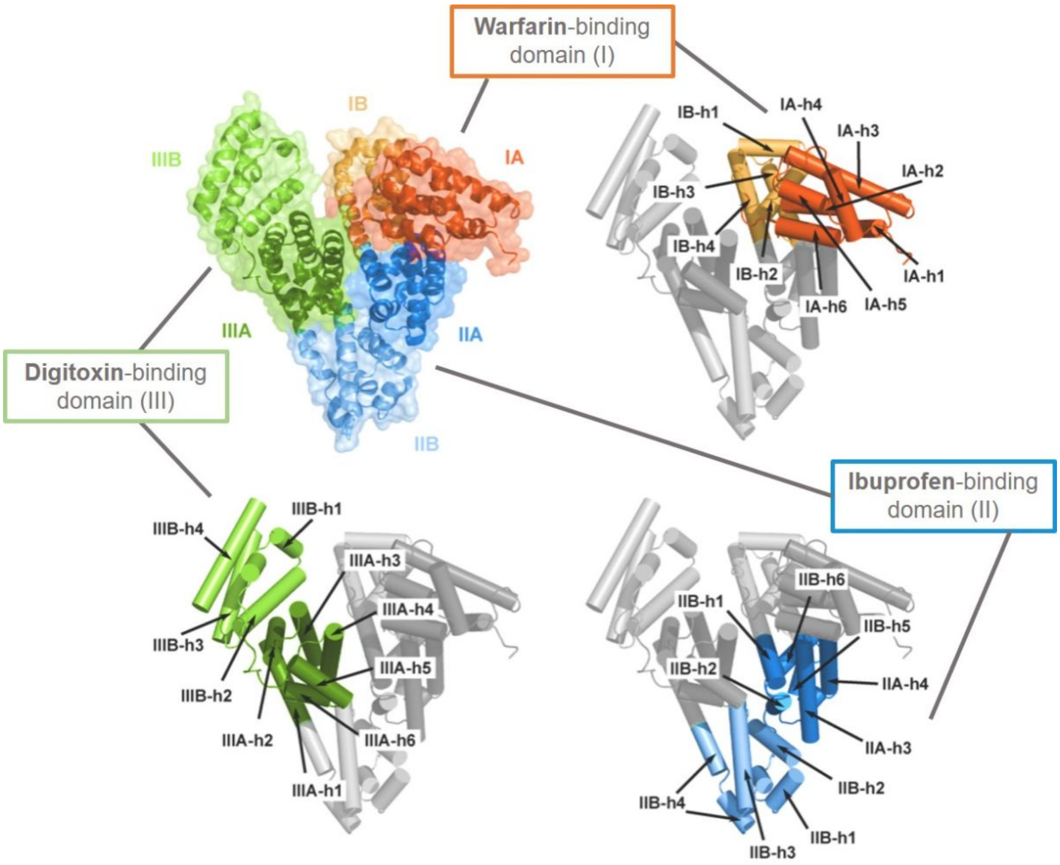


**Figure S2.** Domains and secondary structure elements of the chain A (likewise B) of BSA. Each domain is marked with a different color [orange for the domain I (IA-IB), blue for the domain II (IIA-IIB) and green for the domain III (IIIA-IIIB)] a); each subdomain is highlighted with a different shade, b), c), d). The binding domains of the three clinically-established drugs Warfarin, Ibuprofen and Digitoxin are here emphasized. [readapted from ref. 1]

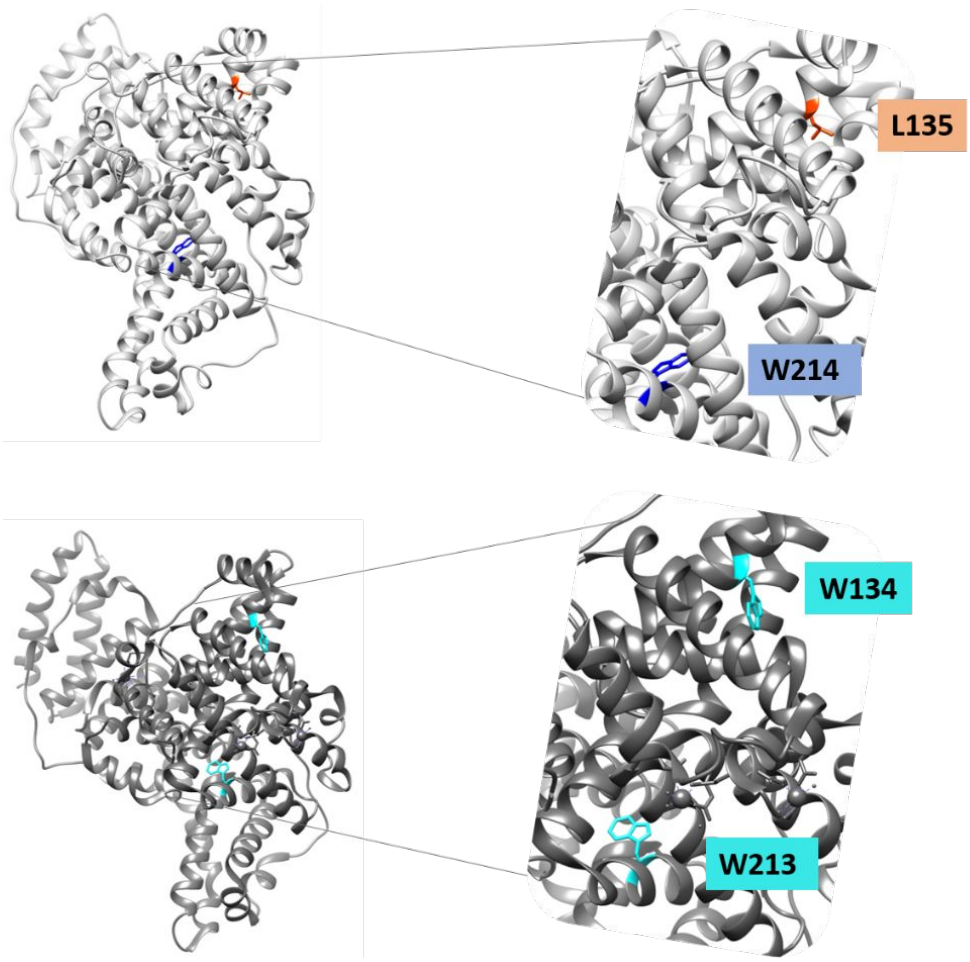


**Figure S3.** Comparison between the chains A of HSA (top) and BSA (bottom), highlighting the presence of one tryptophan residue (W214 – buried in the domain II) and two ones (the buried W213 and W134 on the protein surface in the domain I), respectively. In HSA a Leucine residue (L135) is present instead of a tryptophan one.

This molecular analysis/graphics was performed with UCSF Chimera, developed by the *Resource for Biocomputing, Visualization, and Informatics* at the University of California, San Francisco, with support from NIH P41-GM103311 [http://www.rbvi.ucsf.edu/chimera; UCSF Chimera -- a visualization system for exploratory research and analysis].[2]

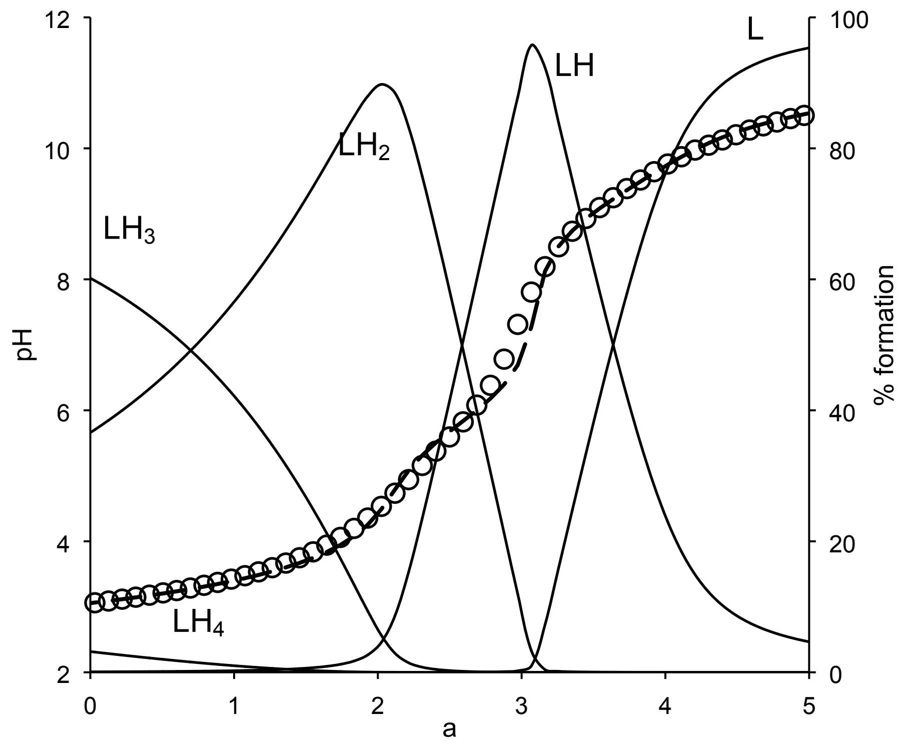


**Figure S4.** Experimental (**O**) and calculated (dashed line) pH values in the potentiometric titration of the b*iso*Qcd ligand (L) (T= 298.2K and μ=0.1M NaCl); [b*iso*Qcd]= 0.02mM); a = (added mol $OH^-$) / (mol L). Charges omitted for clarity.

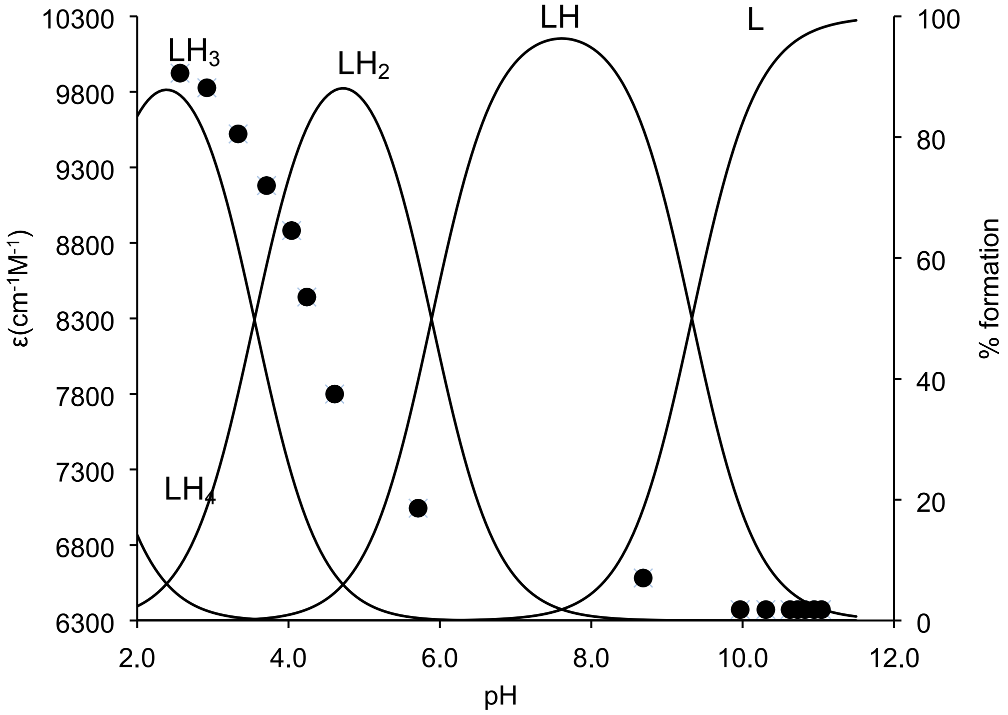

**Figure S5**. Species distribution of the ligand b*iso*Qcd with molar absorbance values at λ= 334 nm obtained by acid-base spectrophotometric titration (298.2K, μ = 0.1 M NaCl). The speciation was calculated using the fitted protonation constants (Table 1).

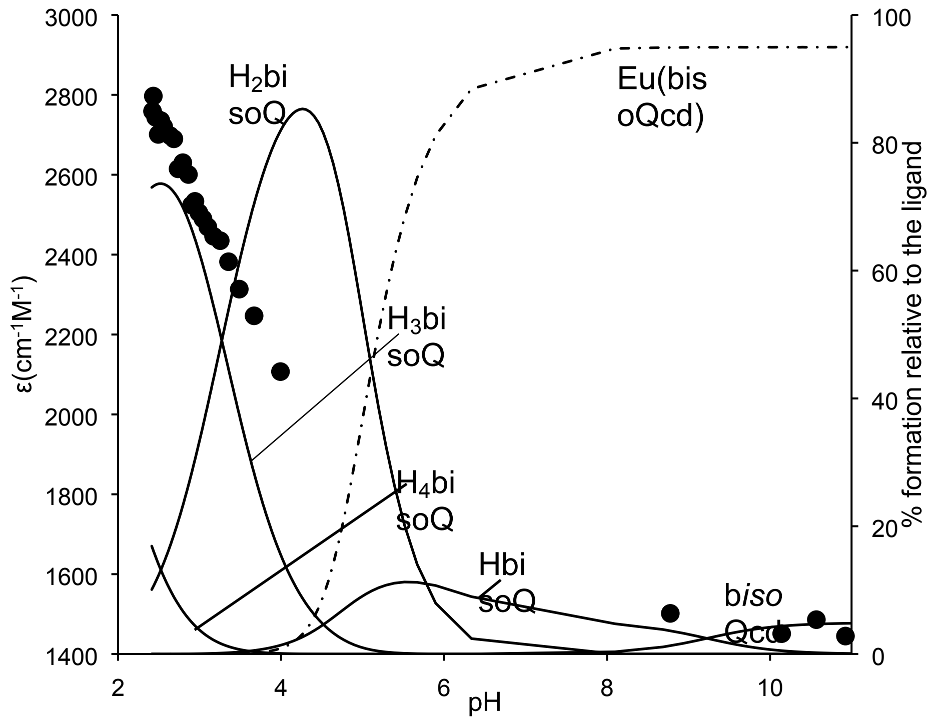


**Figure S6.** Species distribution of the complexes b*iso*Qcd with Eu(III) (M:L ratio 1:1), obtained by acid-base spectrophotometric titration (T= 298.15K). Molar absorbance values at λ = 334nm (●) are also reported.

**Table S1.** Y(III)-N bond distances (Å) for the $[Y(ligand)(H_2O)_2]^+$ complexes reported in Figure 4.

| ligand | Y-$N_{hetero}$ | Y-$N_{amine}$ |
|---|---|---|
| *trans*-O,O-bisoQcd | 2.526 | 2.577 |
| *trans*-N,N-bisoQcd | 2.534 | 2.578 |
| *trans*-O,O-bQcd | 2.666 | 2.567 |
| *trans*-N,N-bQcd | 2.614 | 2.565 |
| *trans*-O,O-bpcd | 2.527 | 2.578 |
| *trans*-N,N-bpcd | 2.525 | 2.575 |

(a)

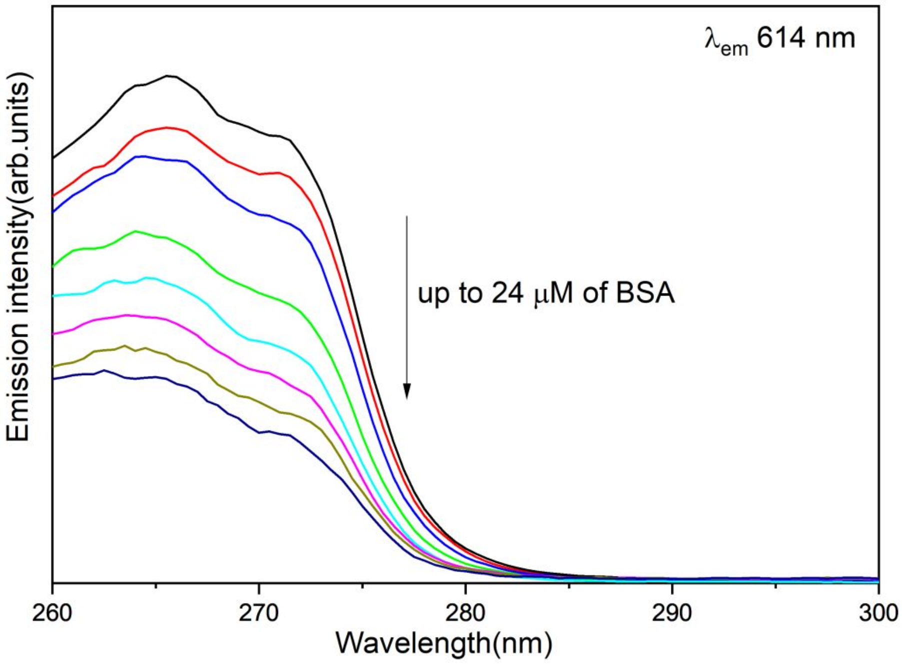


(b)

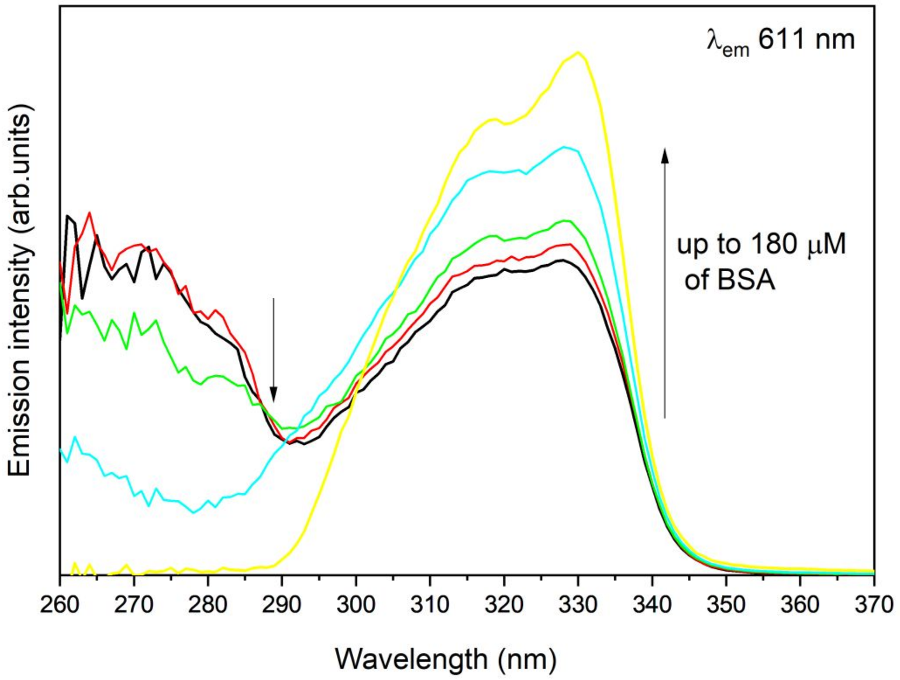


**Figure S7.** Excitation spectra of Eu(III) for: (a) [Eu(bpcd)($H_2O$)$_2$]Cl complex (80 μM) in MOPS-buffered solution (pH 7.4) upon addition of BSA in the 0-24 μM concentration range ($\lambda_{em}$ = 614 nm) and (b) [Eu(b*iso*Qcd)($H_2O$)$_2$]OTf complex (80 μM) upon addition of BSA in the 0-180 μM concentration range at 298 K ($\lambda_{em}$ = 611 nm).

(a)

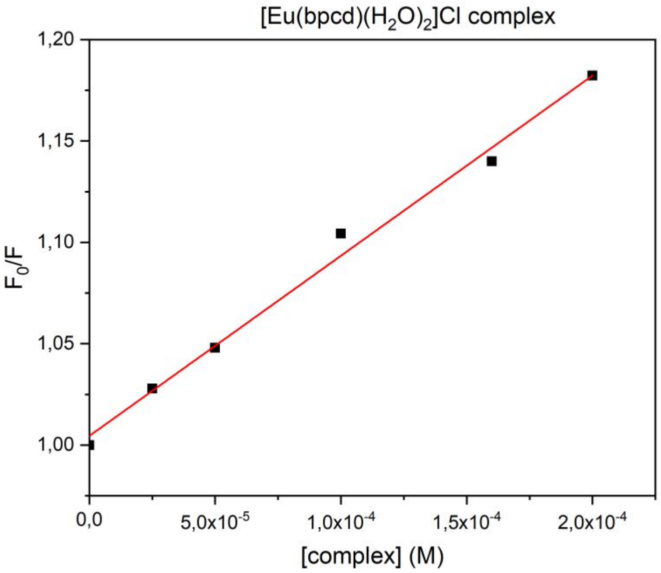


(b)

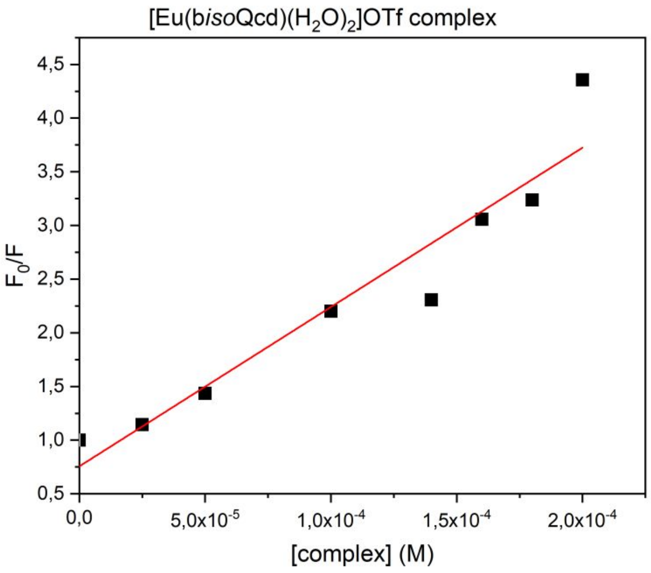


**Figure S8**. Stern-Volmer plot related to the change of the BSA fluorescence upon titration with [Eu(bpcd)($H_2O$)$_2$]Cl (a) and [Eu(bisoQcd)($H_2O$)$_2$]OTf (b). In the case of [Eu(bisoQcd)($H_2O$)$_2$]OTf complex, the Stern-Volmer plot deviates significantly from the linear trend (R-square 88%) and the estimated $K_{sv}$ value is affected by a big error. For this reason, the analysis of BSA fluorescence data upon titration with this complex are no longer discussed following the Stern-Volmer treatment but only using MS-Excel cEST macro program.

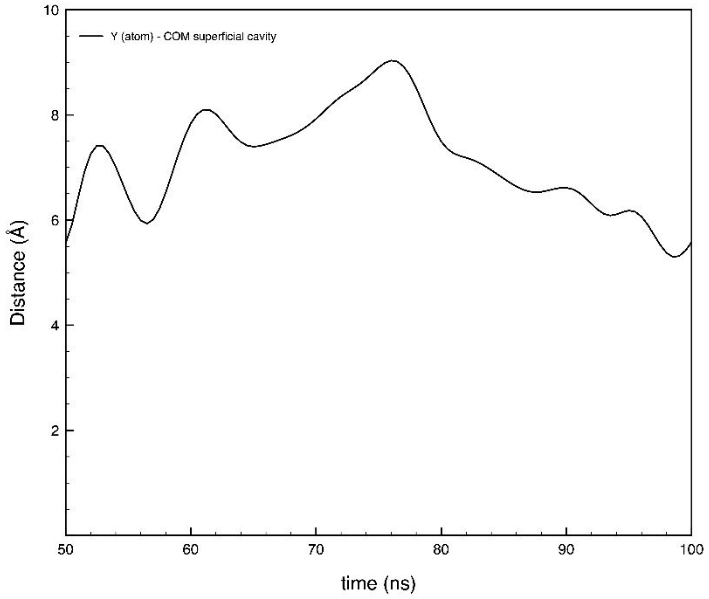

**Figure S9.** Graphical representation of the distance between the Y atom of $[Y(bpcd)(H_2O)_2]^+$ complex and the center of mass (COM) of the residues of the superficial cavity. These residues were selected according to a cutoff of 10 Å from the Y atom. The initial 50 ns were not shown for the sake of clarity, as the molecule explores the entire protein's surface.

(a)

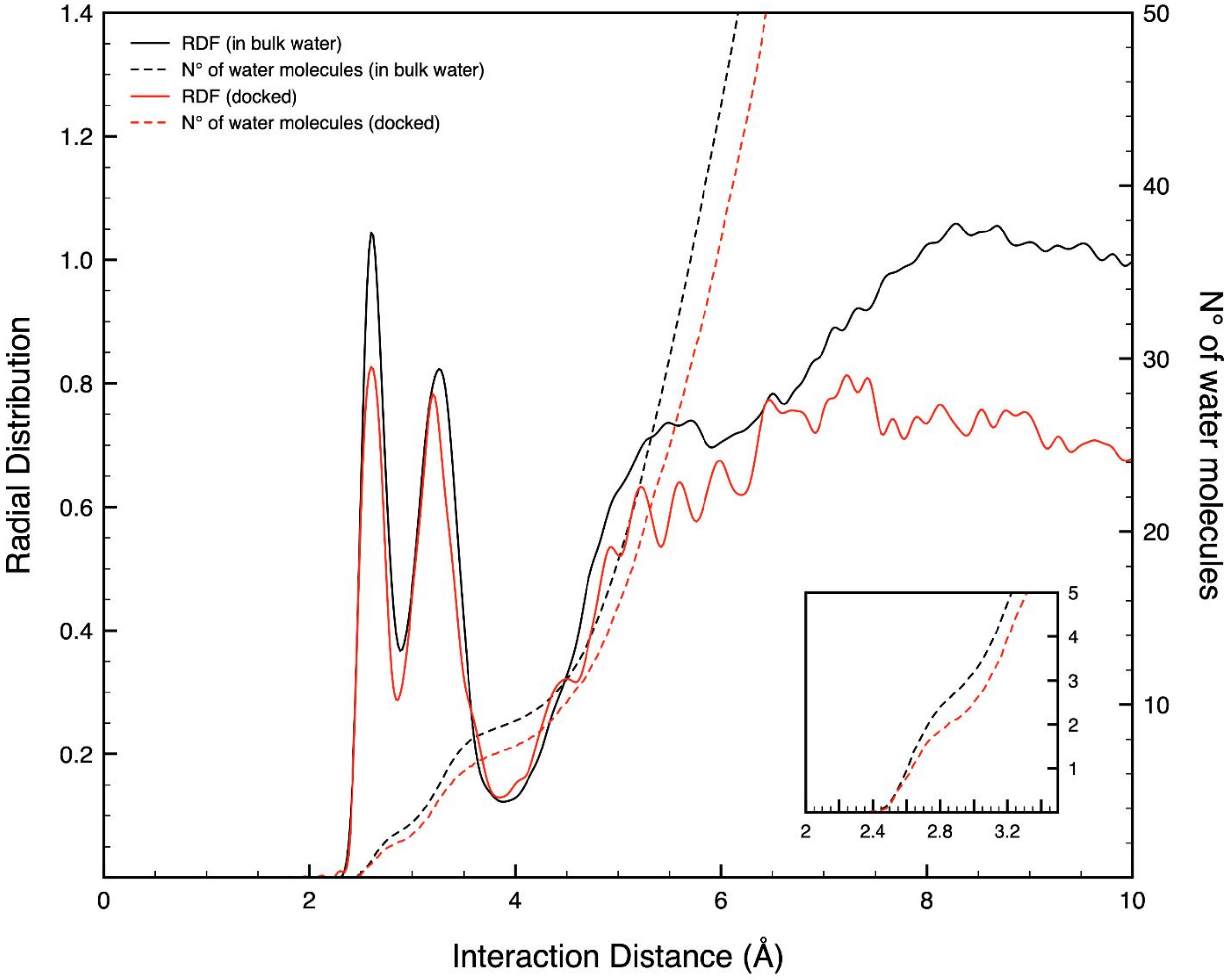


(b)

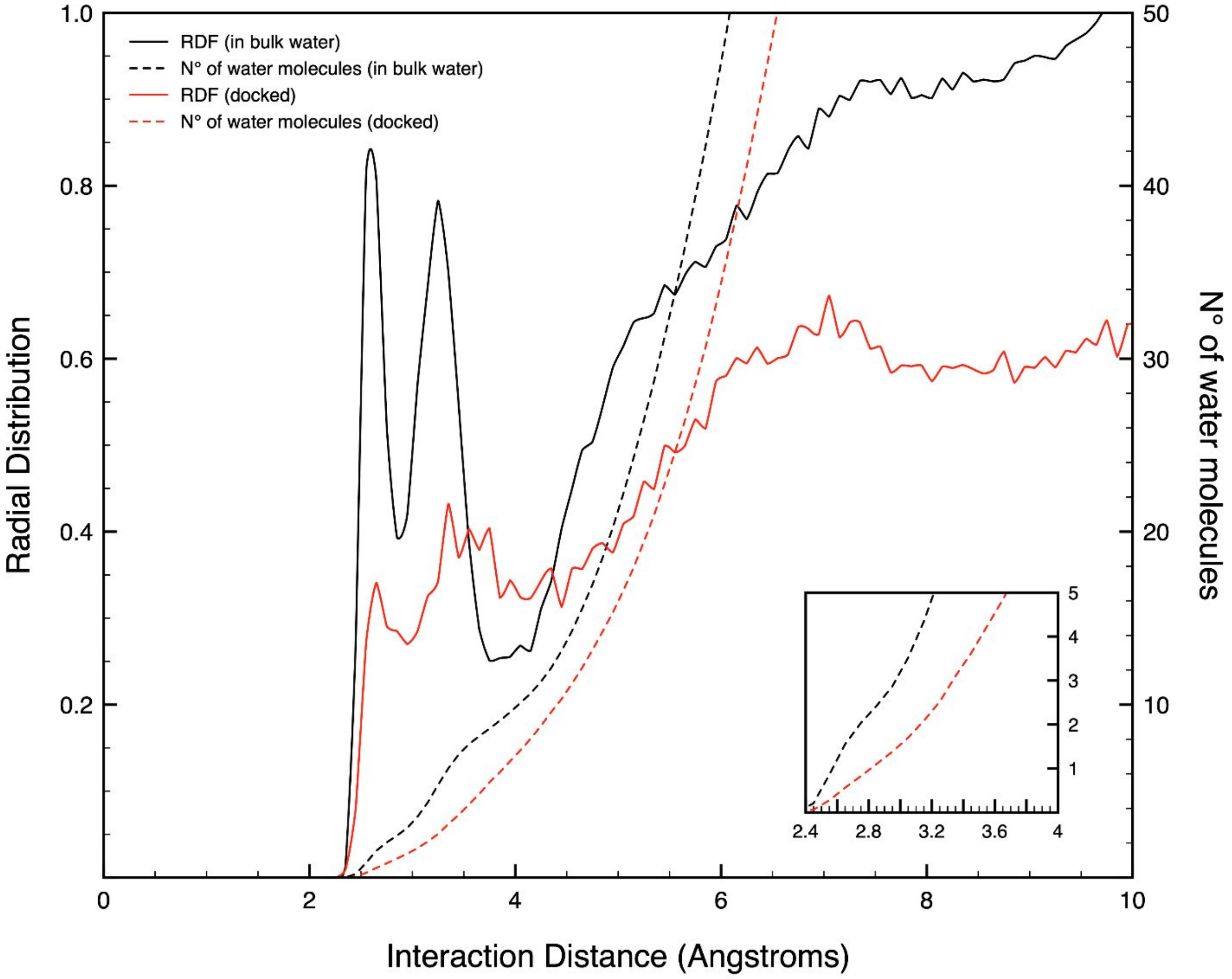


**Figure S10.** Graphical representation of the radial distribution function and the number of water molecules as a function of the interaction distance between Y(III) and water molecules during a simulation in bulk water and upon interaction with BSA, black and red lines, respectively; for the complex with bpcd as a ligand (a) and b*iso*Qcd counterpart (b).

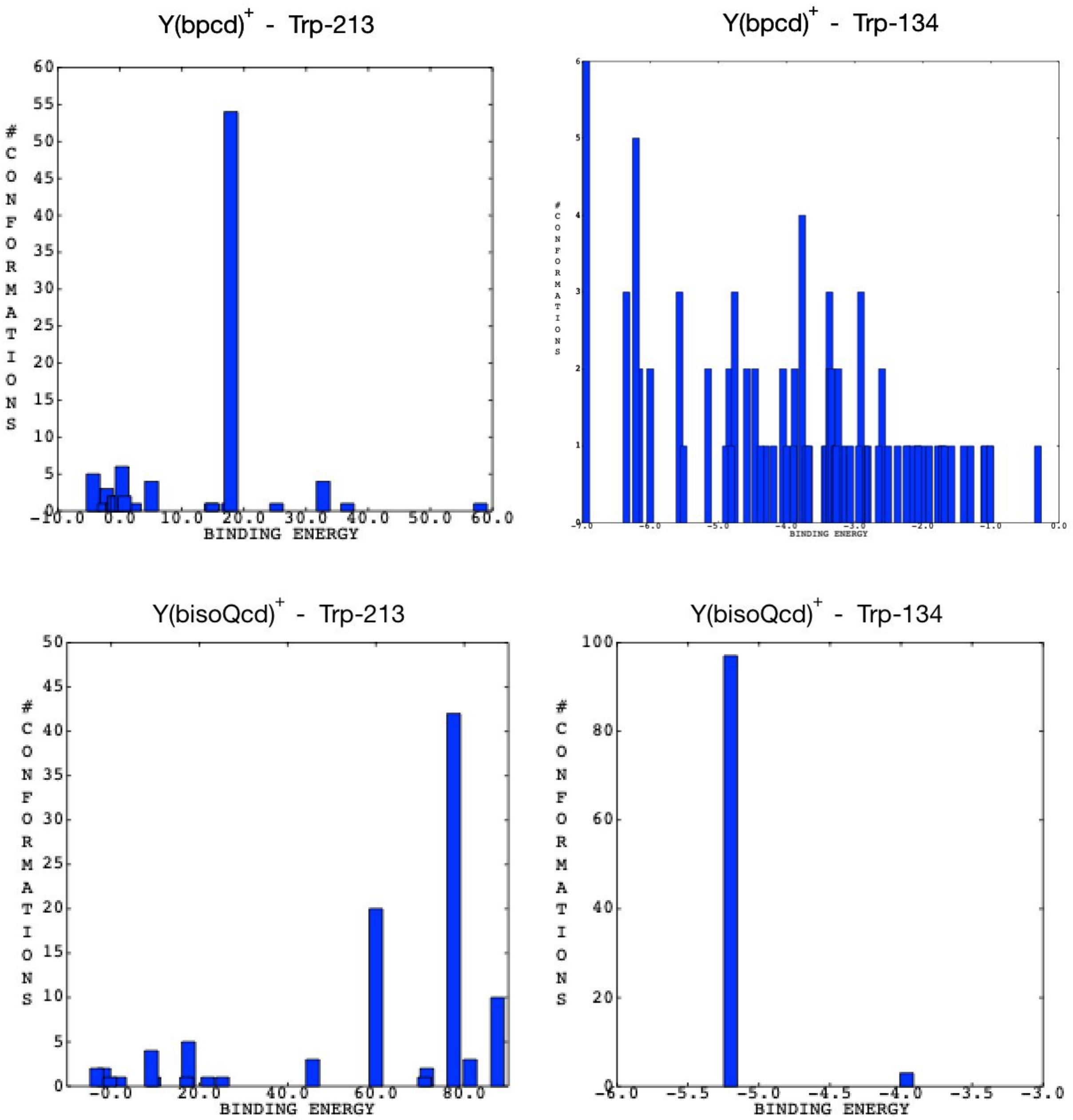


**Figure S11**. Autodock cluster analysis of binding energy between the metal complexes and the BSA protein, at the sites containing the Trp residues.

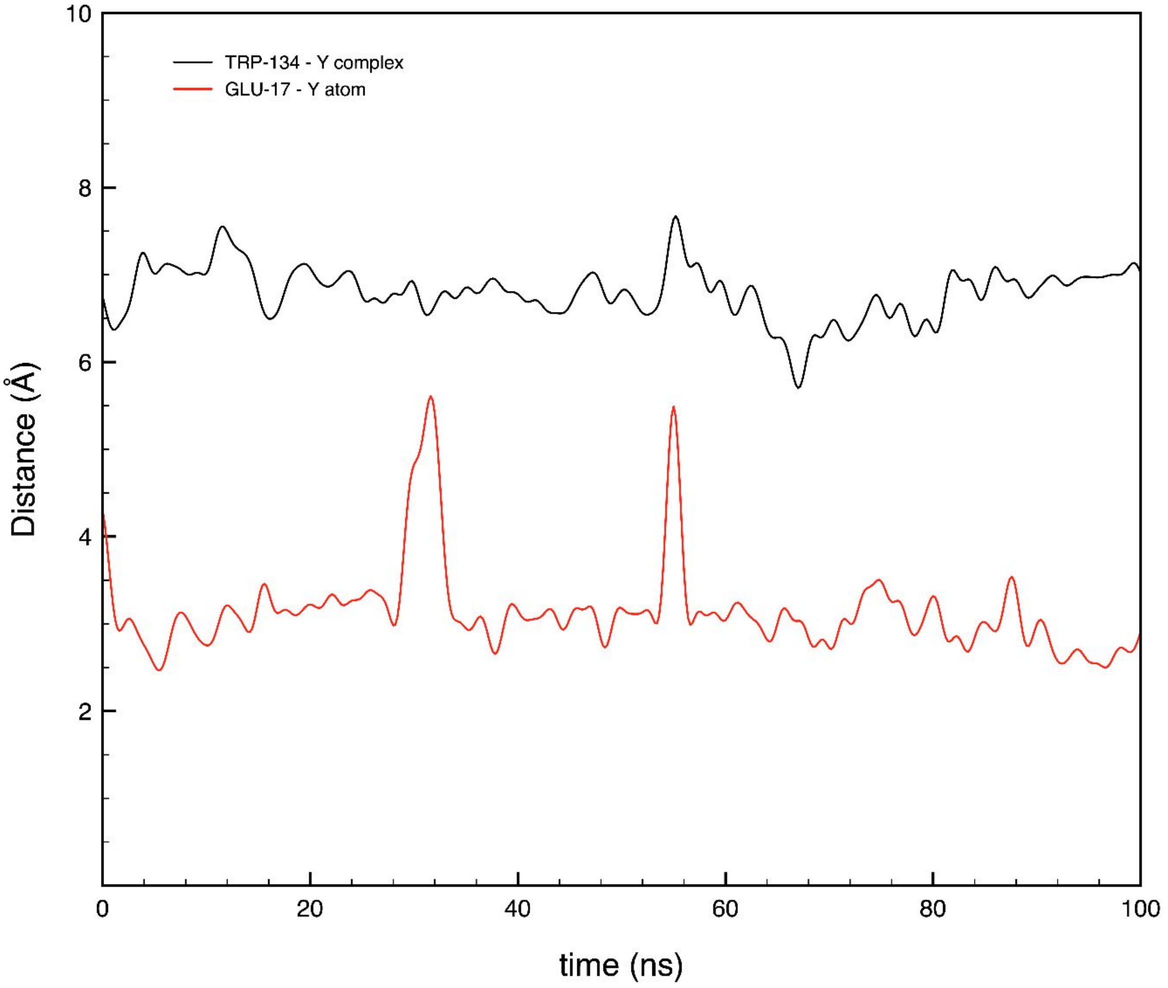


**Figure S12.** Graphical representation of the distance between the side chain of Trp134 and the Y(III) (black plot), and E17 and Y(III) (red plot), in the case of $[Y(bisoQcd)(H_2O)_2]^+$ complex.

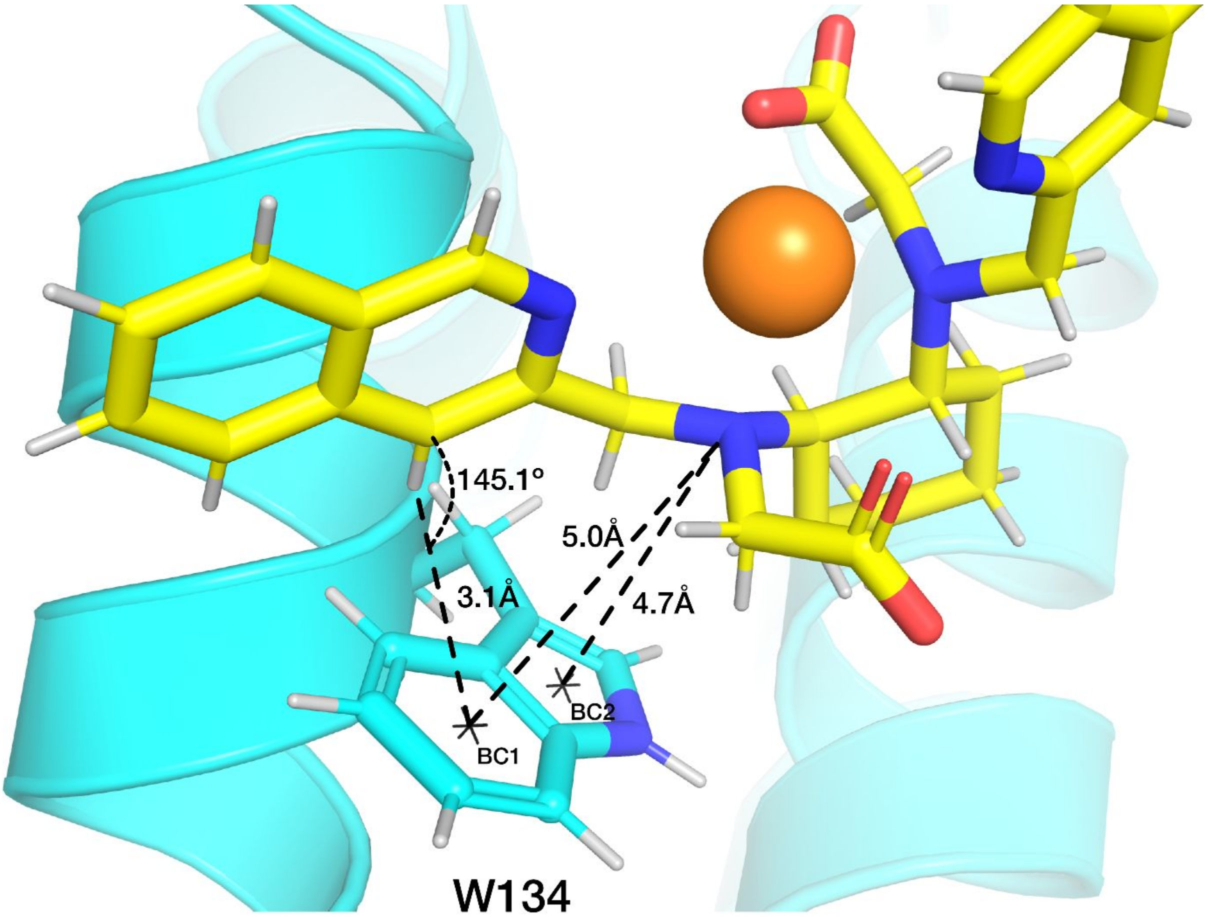


**Figure S13**. Structural detail of the "CH/π interaction" involving Trp134 of BSA and one *iso*quinoline ring of Y(b*iso*Qcd)$^{+}$ complex.

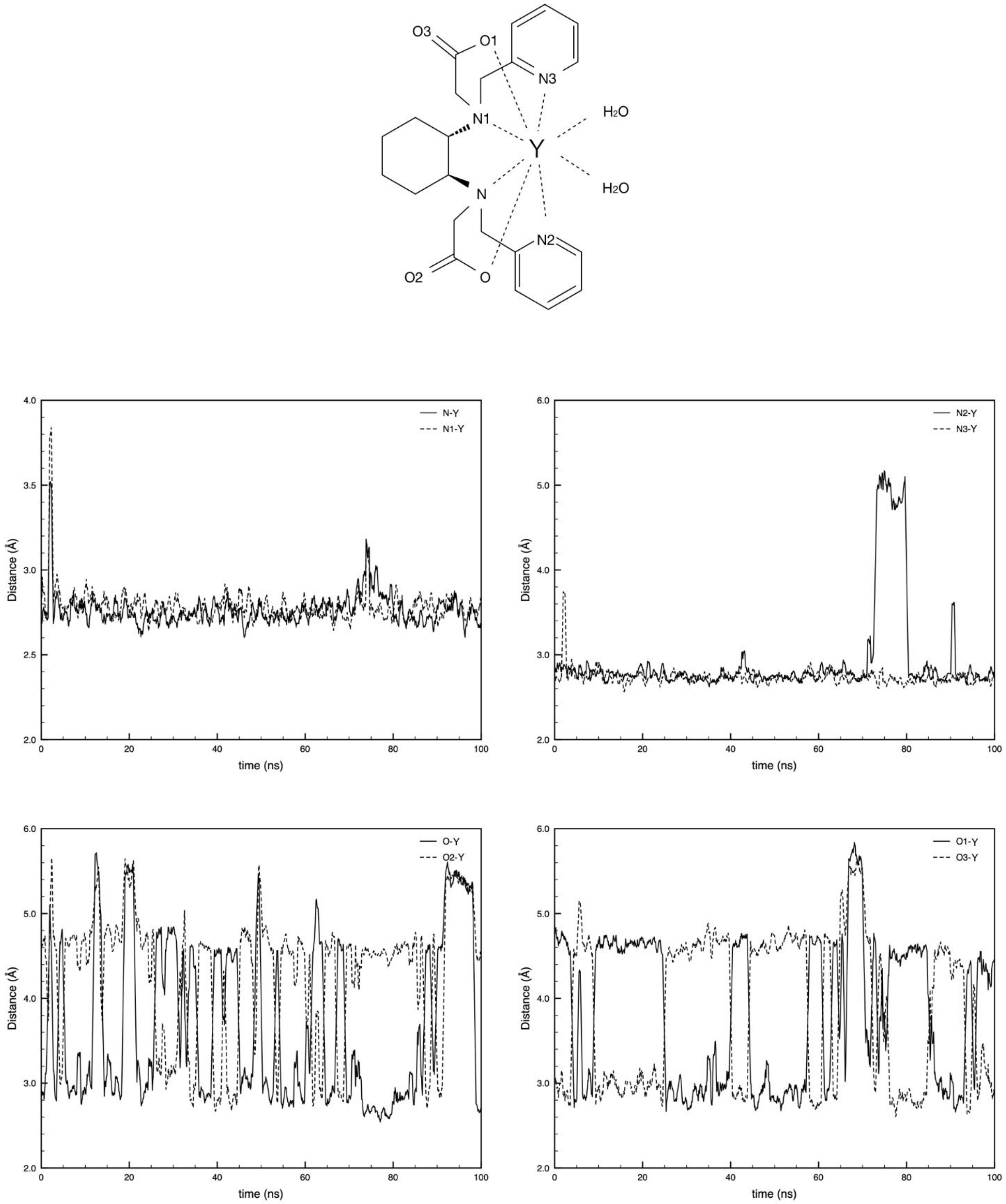


**Figure S14**. Coordination distances of $[Y(bpcd)(H_2O)_2]^+$ upon interaction with the protein.

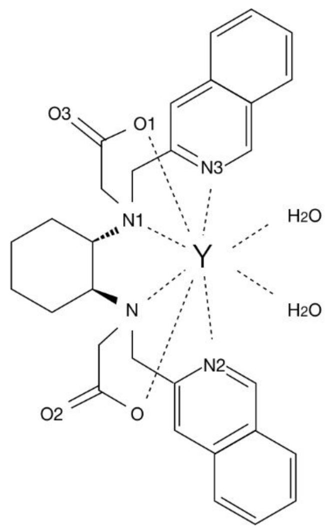

O3
O1
N3
N1
H2O
Y
N
H2O
N2
O2
O


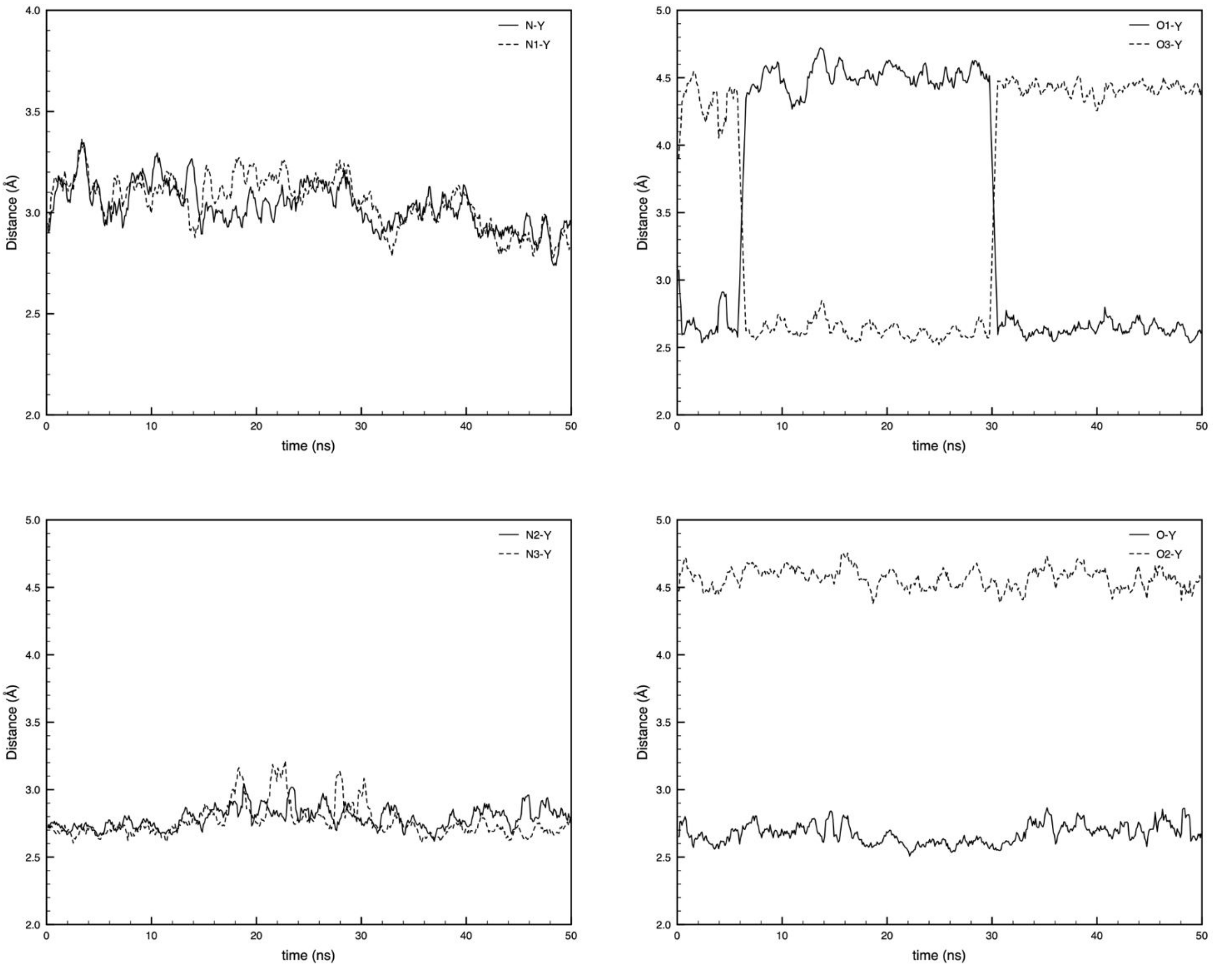

N-Y
N1-Y
O1-Y
O3-Y
N2-Y
N3-Y
O-Y
O2-Y
Distance (Å)
time (ns)

**Figure S15**. Coordination distances of [Y(b*iso*Qcd)($H_2O$)$_2$]$^+$ upon interaction with the protein.

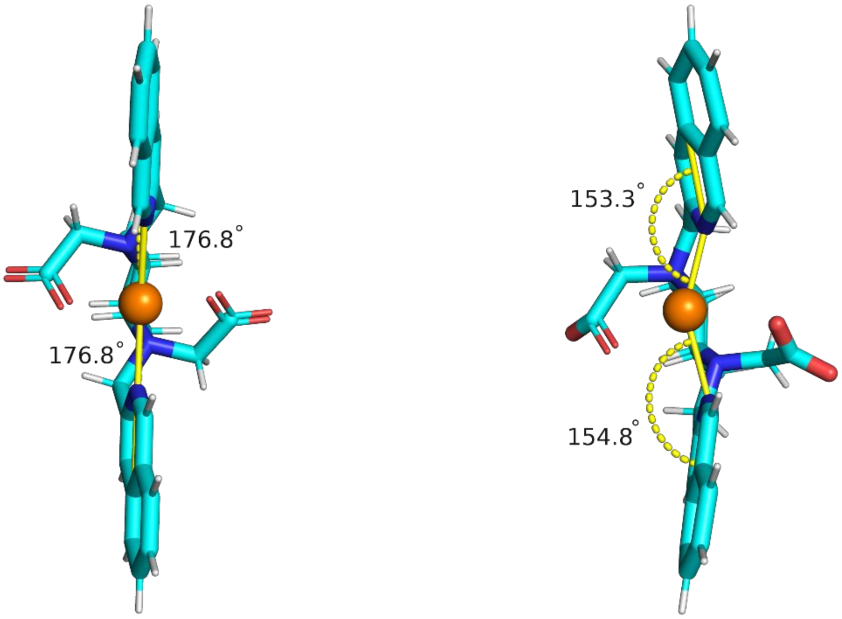


**Figure S16**. Representation of the geometry before (left) and after (right) the interaction with BSA for the [Y(b*iso*Qcd)($H_2O$)$_2$]$^+$ complex

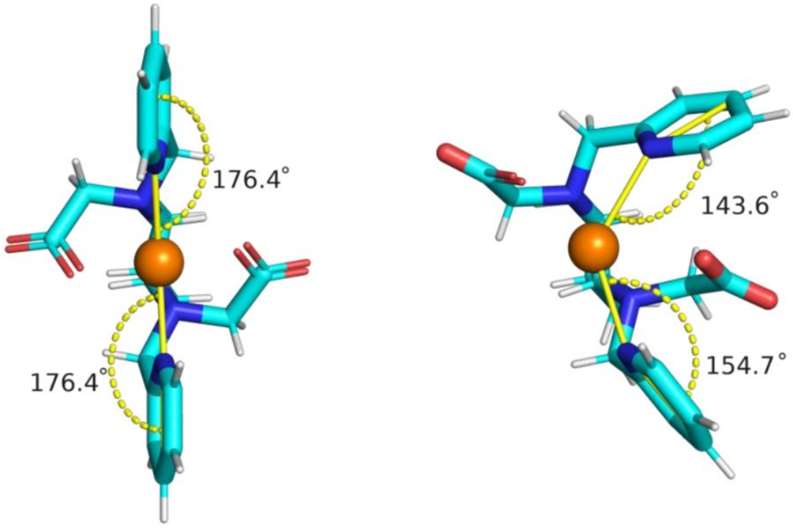


**Figure S17**. Representation of the geometry before (left) and after (right) the interaction with BSA for the [Y(bpcd)($H_2O$)$_2$]$^+$ complex .

## References


(1) Majorek, K. A.; Porebski, P. J.; Dayal, A.; Zimmerman, M. D.; Jablonska, K.; Stewart, A. J.; Chruszcz, M.; Minor, W. Structural and Immunologic Characterization of Bovine, Horse, and Rabbit Serum Albumins. *Mol. Immunol.* **2012**, *52*, 174–182.

(2) Pettersen, E. F.; Goddard, T. D.; Huang, C. C.; Couch, G. S.; Greenblatt, D. M.; Meng, E. C.; Ferrin, T. E. UCSF Chimera - A Visualization System for Exploratory Research and Analysis. *J. Comput. Chem.* **2004**, *25*, 1605–1612.
